# Imitating and exploring the human brain's resting and task-performing states via brain computing: scaling and architecture

**DOI:** 10.1093/nsr/nwae080

**Published:** 2024-03-01

**Authors:** Wenlian Lu, Longbin Zeng, Jiexiang Wang, Shitong Xiang, Yang Qi, Qibao Zheng, Ningsheng Xu, Jianfeng Feng

**Affiliations:** Institute of Science and Technology for Brain-Inspired Intelligence, Fudan University, Shanghai 200433, China; Key Laboratory of Computational Neuroscience and Brain-Inspired Intelligence (Fudan University), Ministry of Education, Fudan University, Shanghai 200433, China; Shanghai Center for Mathematical Sciences, Fudan University, Shanghai 200433, China; Institute of Science and Technology for Brain-Inspired Intelligence, Fudan University, Shanghai 200433, China; Institute of Science and Technology for Brain-Inspired Intelligence, Fudan University, Shanghai 200433, China; Institute of Science and Technology for Brain-Inspired Intelligence, Fudan University, Shanghai 200433, China; Key Laboratory of Computational Neuroscience and Brain-Inspired Intelligence (Fudan University), Ministry of Education, Fudan University, Shanghai 200433, China; Institute of Science and Technology for Brain-Inspired Intelligence, Fudan University, Shanghai 200433, China; Key Laboratory of Computational Neuroscience and Brain-Inspired Intelligence (Fudan University), Ministry of Education, Fudan University, Shanghai 200433, China; Institute of Science and Technology for Brain-Inspired Intelligence, Fudan University, Shanghai 200433, China; Key Laboratory of Computational Neuroscience and Brain-Inspired Intelligence (Fudan University), Ministry of Education, Fudan University, Shanghai 200433, China; Institute of Science and Technology for Brain-Inspired Intelligence, Fudan University, Shanghai 200433, China; Institute of Science and Technology for Brain-Inspired Intelligence, Fudan University, Shanghai 200433, China; Key Laboratory of Computational Neuroscience and Brain-Inspired Intelligence (Fudan University), Ministry of Education, Fudan University, Shanghai 200433, China; Shanghai Center for Mathematical Sciences, Fudan University, Shanghai 200433, China; Department of Computer Science, University of Warwick, Coventry CV4 7AL, UK; Zhangjiang Fudan International Innovation Center, Fudan University, Shanghai 200433, China

**Keywords:** digital twin brain, computational cortico-subcortical model, fMRI, data assimilation, interoceptive circuit

## Abstract

A computational human brain model with the voxel-wise assimilation method was established based on individual structural and functional imaging data. We found that the more similar the brain model is to the biological counterpart in both scale and architecture, the more similarity was found between the assimilated model and the biological brain both in resting states and during tasks by quantitative metrics. The hypothesis that resting state activity reflects internal body states was validated by the interoceptive circuit's capability to enhance the similarity between the simulation model and the biological brain. We identified that the removal of connections from the primary visual cortex (V1) to downstream visual pathways significantly decreased the similarity at the hippocampus between the model and its biological counterpart, despite a slight influence on the whole brain. In conclusion, the model and methodology present a solid quantitative framework for a digital twin brain for discovering the relationship between brain architecture and functions, and for digitally trying and testing diverse cognitive, medical and lesioning approaches that would otherwise be unfeasible in real subjects.

## Teaser

This paper presents a solid framework of digital twin brain (DTB) for discovering the relationship between brain architecture and functions, and digitally trying and testing diverse cognitive and medical approaches that are infeasible to be examined in biological subjects.

## INTRODUCTION

It has been widely believed that simulating brain dynamics is a promising way to understand the functions of the human brain, particularly high cognitive functions, as well as their causal relations to brain structures and dynamics [1], and that it may lead to the next generation of artificial intelligence (AI) [2], as it did at the beginning of AI development [3,4]. However, simulating human brain dynamics has always been extremely difficult due to many factors. First, the scale of the human brain, with up to 86 billion neurons and 100 trillion synapses [5,6], including long-distance and irregular connections, creates great challenges for both high-performance computing systems [7–11] and brain-inspired chips [12–16] to simulate a brain model of human-brain scale with low time-to-solution and cost. Second, despite progress in simulating the human brain with limited structural information, the fine structures and activities of the whole human brain at neuronal and synaptic levels cannot be observed *in vivo* and as a result, dynamical models are always based on brain regions [17–19].

More importantly, assimilating human brain functional data from computing models obtained from structural data is a way to show similarity in the functional connectivity (FC) and time courses of brain imaging, including functional magnetic resonance imaging (fMRI), electroencephalography (EEG) and magnetoencephalography (MEG), between the computing model and its biological counterpart [20–22]. Statistical physical methods have been used to identify the physical properties of brain dynamics and their relationship to brain functions [23]. However, most of these works use a dynamical model based on neuron populations or brain areas. Related to this work, we have developed a framework of data assimilation to statistically infer parameters in a large-scale neuronal network model by limited data through a hierarchical Bayesian method [24], with sufficient computing power. We are at a stage where we can explore the human brain and its functions via the computing brain model at the neuron level and human brain scale, with the help of sufficient computing power. However, the validity and rationality of assimilating such a ‘big’ brain model *in silico*, with necessary costs, need to be demonstrated. More generally, the digital twin brain (DTB) concept and technology were introduced to produce a digital representation of the structure, computing processes and intelligent functions of a biological brain [25]. This will provide a platform for digitally testing diverse cognitive and medical approaches that cannot be carried out on a real biological subject.

In this research article, we present a framework to investigate both resting-state and task-performing dynamics of the computing model at the neuron level and human cortico-subcortical scale, with up to 20 billion neurons, by developing a voxel-wise diffusion hierarchical mesoscale data assimilation (Vw-dHMDA) method to assimilate multimodal neuroimaging data, including structural MRI, diffusion-weighted MRI, positron emission tomography (PET) and fMRI. We have found that the similarities between the model and the biological counterpart, measured by the similarity in FC, time courses of blood-oxygen-level-dependent (BOLD) signals, as well as avalanche criticality and synchrony of brain dynamics, increase with the scale of the computing model in terms of the number of neurons and the average number of synaptic connections, both in resting state and during tasks. Moreover, in the evaluation tasks, the predicted task scores of the model are more similar to those of its experimental counterpart as the model scale increases. In addition, the roles of subcortices in the brain's resting state have been explored by the assimilating framework. This cortico-subcortical model gives us exciting computing tools to investigate and validate theories of causal relations between the subcortical structures and dynamical functions, by digitally manipulating the network structure of computing models. We have found that the similarity may relatively decrease when the model architecture is gradually rewired from the DWI-based network to a locally connected graph in both resting and task-performing states, and may also decrease at the hippocampus, when removing the connections from the primary visual cortex (V1) to the dorsal and/or ventral visual pathways, in comparison to the motor pathway, during tasks. We highlight that one can benefit from the digital brain models and the associated statistical methods because these investigations and operations cannot be carried out in real human brains.

## RESULTS

### Computational cortico-subcortical model with flexible scaling

We utilized the multimodal imaging data, including structural MRI (T1 weighted images) and multi-shell diffusion-weighted images (DWI) [25], of a single subject, denoted by the SUBJECT in the present paper, combined with the synaptic vesicle glycoprotein 2A (SV2A) PET images for neuronal synaptic density for all voxels, and a laminar model [26,27] for all cortical voxels, to establish the computational neuronal network model for the cortex and subcortex.

**Figure 1.**
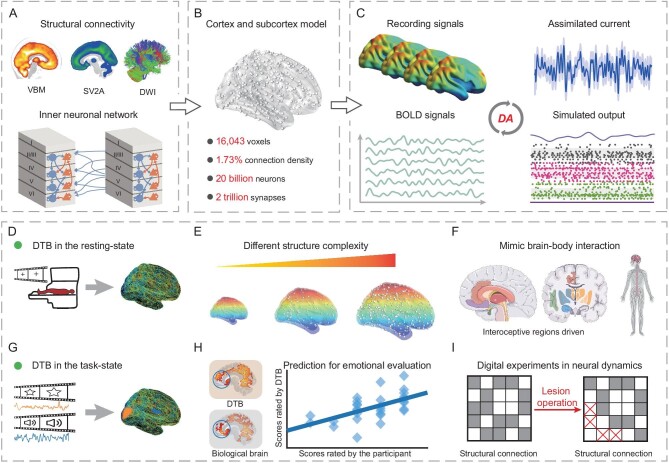
Workflow of the cortico-subcortical model (similar to the DTB model [11]). (A) The computational cortico-subcortical model is constructed via a collection of biological data (VBM, SV2A and DWI) and neurons in each voxel of the cortex are organized into a micro-column structure. (B) The cortico-subcortical network is sampled according to the assigned numbers of neurons in each population (layer or voxel) and their connection probabilities, and the model contains 374 brain regions, 16 043 voxels, totalling 20 billion neurons and 2 trillion synapses with 1.73% connection density. (C) This model is updated sequentially with the Vw-dHMDA method in real time. Then the simulated output (firing rate) is fed into the Ballon-Windkessel model to generate the time series of the simulated blood-oxygenation-level-dependent (BOLD) signal. The external current is tuned to fit the empirical BOLD signal from experimental recording (fMRI). (D, G) The basic numerical experiments are centred on simulating biologically plausible networks in resting states and during tasks. (E) More resemblance implies more similarity. We perform a series of experiments with different network scales and different levels of structural destruction on DWI-based architecture to verify the biological similarity between the digital brain and its real counterpart in resting states. (F) We also explore the effect of interoceptive regions on network dynamics. (H) The prediction performance based on the assimilated cortico-subcortical model for the auditory and visual evaluation tasks is also tested with different network sizes. (I) We remove the ventral and dorsal pathways of visual perception to study the importance of network structure on task performance.

Following the pipeline of the DTB [11,25], as shown in Fig. 1, the neuronal network model is flexible concerning the number of neurons and the average synaptic connection in-degree, that is, the average number of synapses received by each neuron. Generally speaking, in this network architecture, the number of neurons in each voxel is proportional to the gray matter volume determined by voxel-based morphometry (VBM), the synaptic density for each voxel is proportional to the SV2A data [28,29] acquired from PET imaging, and the number of the excitatory synaptic connections between two voxels is proportional to the track density measured by normalized DWI (Fig. 1A). In particular, each cortical voxel is modelled with a laminar structure consisting of L2/3, L4, L5 and L6, with the distribution of the number of neurons in each layer and the excitatory and inhibitory inter-layer synaptic connections following the laminar model data (Fig. 1A). The full description of the model architecture can be found in the Supplementary Data. Therein, we highlight that one can sample a directed neuronal network of diverse numbers of neurons and average synaptic connection in-degrees of 16 043 voxels and 374 regions, with up to 20 billion neurons (Fig. 1B). The hardware and software environments of the simulation and their performance are shown in the Supplementary Data.

In the computational model, each neuron is represented by the leaky integrate-and-fire (LIF) model equipped with two types of synapses (i.e. AMPA and GABA). The response of each of these synapses to an input spike exhibits an instantaneous jump followed by an exponential decay. We consider a background current injected into each neuron as driven by an Ornstein–Uhlenbeck (OU) process. Hence, with the neuronal activities (such as spikes and corresponding firing rates) obtained from the computational model, the simulated BOLD signal for each neuronal population (voxel) is then obtained using the Balloon-Windkessel model [30].

In certain regions, each neuron in a voxel is injected with an external current, which follows a Gamma distribution with the same hyperparameter as the other neurons in the same voxel. These voxel-wise hyperparameters can be estimated by experimental BOLD signals in both resting state and during tasks of the same subject, using the Vw-dHMDA method (Fig. 1C). Then, by sampling the current from the estimated hyperparameter, the simulated BOLD signals can be generated. Herein, the performance of this statistical inference is evaluated by the similarities in the time course of BOLD signals and the region-wise functional connectivity between the simulated data and their biological counterparts.

### Scaling investigation of resting states

**Figure 2.**
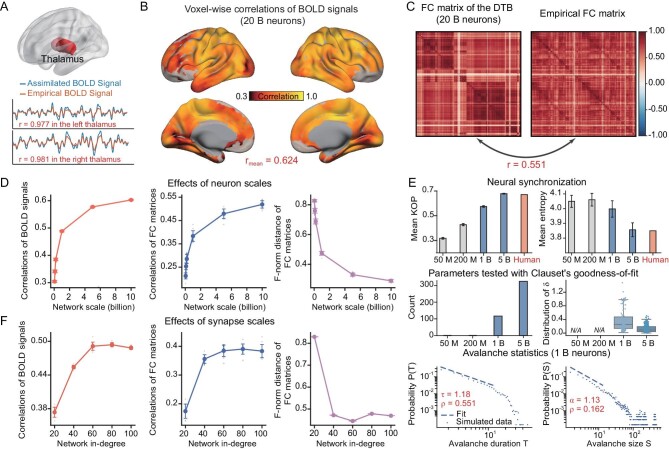
Scaling analysis of the cortico-subcortical model in the resting state. (A) Upper panel: schematic representation of the resting-state experiments driven by the thalamus. Lower panels: correlations of BOLD signals in the thalamus. (B) Voxel-level Pearson correlation coefficients (PCCs) between the empirical and assimilated BOLD signals, with the latter generated by the model with 20 billion neurons over all voxels. (C) Illustration of FC of the model with 20 billion neurons and empirical FC. (D) Performance of the computational cortico-subcortical model in the resting state concerning the number of neurons in the network. The evaluation metrics are the PCCs between voxel-wise BOLD signals of the model and those of the real brain, the PCCs between the region-wise FC matrices of the model and those of the real brain, and the F-norm distance between the FC matrices of the model and the data. Ten repetitions of simulation experiments were conducted on models with an average synaptic connection in-degree of 100 and models of 0.05, 0.1, 0.2, 1, 5 and 10 billion neurons. (E) Phase synchrony analysis and avalanche criticality analysis of the DTB in the resting state with diverse numbers of neurons: 0.05, 0.2, 1 and 5 billion. Upper panel: both the means and entropies of Kuramoto order parameters of region-wise BOLD signals are calculated to quantify the mean and variability in synchronization in models with an average synaptic connection in-degree of 100 and a neuron number of 0.05, 0.2, 1 and 5 billion, as compared to the biological brain. The light gray bars correspond to the network size in the middle panel where parameters did not pass Clauset's goodness-of-fit test. Middle panel: number of parameters tested via Clauset's goodness-of-fit test and the corresponding distribution of δ in models with synapse scales consistent with the upper panel. The tested parameters include thresholds and fitting windows for avalanche size S and avalanche duration T. The quantity ${\mathrm{\delta }} = {\mathrm{\gamma }} - |\frac{{1 - \alpha }}{{1 - \tau }}$| represents the distance to the critical point. Lower panel: the probability distributions of avalanche durations and avalanche sizes for the threshold 2.4 SD and the time bin width of 1 volume (vol.) in the assimilated BOLD signals. The distributions of avalanche durations are well approximated by power law with an exponent of τ=1.18 with Clauset's test ρ = 0.551. The distribution of avalanche sizes can be fitted well by a power law with an exponent of α = 1.13 with Clauset's test ρ = 0.162. The fitting windows are ${{T}_{{\mathrm{min}}}} = $2, ${{T}_{{\mathrm{max}}}} = $13 and ${{S}_{{\mathrm{min}}}} = $2, ${{S}_{{\mathrm{max}}}} = $12, respectively. (F) Assessment of DTB performance in the resting state based on the average synaptic connection in-degree, using the same three evaluation metrics as shown in panel (D). Ten repetitions of simulation experiments were conducted on models with network sizes of 1 billion and an average in-degree of 20, 40, 60, 80 and 100.

We first assimilate the resting state of the cortico-subcortical model by fitting the resting-state BOLD signals of the voxels in the thalamus region, which is regarded as a relay spot, by estimating the voxel-wise hyperparameters of the input currents injected to the neurons, in a neuronal network of 2 billion neurons with an average synaptic connection in-degree of 100, using the structure mentioned above. The average Pearson correlation coefficient (PCC) between the simulated and experimental BOLD signals over all voxels at the thalamus is 0.977 (left) and 0.981 (right) (Fig. 2A). Then, we can simulate the neuronal network models driven by the left and right thalamus (with the assimilated time series of the external current hyperparameter) (Fig. 1D). We measure the similarity between the assimilated model and its biological counterpart by calculating the PCCs between the time courses of voxel-wise resting-state BOLD signals of the assimilated model and those of the real brain, and by the similarities of the region-wise FC matrices calculated from the assimilated and biological resting-state BOLD signals, measured by their PCCs and the Frobenius norm (F-norm) of the difference between these two FC matrices. In this way, we achieve significant similarities between the computational cortico-subcortical model, with 20 billion neurons and an average synaptic connection in-degree of 100, and the resting-state fMRI. The average PCC in the BOLD signals over all voxels is 0.624 (Fig. 2B), the PCC between the FC matrices of the simulated model and that of real BOLD signals is 0.551 (Fig. 2C), and the F-norm distance between these FC matrices is 0.271.

We investigate the influence of scale on these similarities between the model and biological data by varying the number of neurons and the average synaptic in-degree (Fig. 1E). It is shown that the similarities between the assimilated resting-state BOLD signals and the biological data significantly increase with the number of neurons, if fixing the average synaptic connection in-degree as 100. The mean PCCs between the assimilated and the biological voxel-wise resting-state BOLD signals increase with the number of neurons. The PCCs of these two FC matrices increase with the number of neurons and the F-norm distance between these two FC matrices decreases as the number of neurons increases (Fig. 2D) as well.

The similarities between the assimilated and biological resting-state BOLD signals increase with the average synaptic connection in-degree, with the number of neurons set to 1 billion. By the same metrics, the average voxel-wise PCCs between the simulated and biological resting-state BOLD signals first increase and then stay stable with the average synaptic connection in-degree, with the number of neurons set to 10 billion; the PCC in the region-wise FC matrices between the simulated and biological resting-state BOLD signals increases, and the F-norm distance between these two FC matrices decreases with the average in-degree as well (Fig. 2F). Similar results hold if the neuronal connection follows various in-degree distributions, as we have demonstrated in Fig. S10, provided that the variance of the degree distribution is not extremely large.

The critical brain hypothesis suggests that efficient neural computation can be achieved through critical dynamics and that the brain operates in close vicinity to a critical point lying between order and disorder [23]. This hypothesis is supported by a set of observations of power-law scaling in many different neural systems using various approaches [31,32]. The avalanche criticality analysis shows that the assimilated region-wise resting-state BOLD signals of a series of neuronal networks of 1 billion neurons, sampled from the biological structural data, is near to a critical point, with the avalanche duration and size close to power-law distributions, with the exponents found to be 1.18 and 1.13 respectively (Supplementary Data and Fig. 2E bottom panel).

When the number of neurons is below 1 billion (0.05 and 0.2 billion), the assimilated resting-state BOLD signals are far away from the critical point since the avalanche durations and sizes did not follow the power-law distribution (Fig. 2E middle panel). Moreover, in comparison, when the number of neurons increases to 5 billion, the simulated resting-state BOLD signals are closer to the critical point than the model of 1 billion neurons, since the avalanche durations and sizes are close to the power-law distribution and its mean $\delta $-value is much smaller (Fig. 2E middle panel).

We also measure the mean and variability in synchronization with a previously described approach [33]. Both the mean synchronization (MS) and synchronization entropy (SE) of region-wise BOLD signals generated by the model get gradually closer to those of the biological resting-state BOLD signals when the number of neurons increases from 0.05 to 5 billion. The mean of the region-wise Kuramoto order parameter increases with the number of neurons and the entropy of the region-wise Kuramoto order parameter decreases with the number of neurons, towards the values calculated by the BOLD time course of the biological data, which are found to be 0.671 for the mean and 3.850 for the entropy (Fig. 2E top panel).

### Architectural rewiring investigation of resting states

Besides the scales, the other significant characteristics of the computational models lie within the dependence on the DWI-based neuroanatomy. To demonstrate the influence of this dependence, we carry out a rewiring process on the neuronal network, with the number of neurons set to 1 billion and with the average synaptic connection in-degree set to 100. Starting with the original voxel-wise network architecture, the rewiring operation involves randomly picking connections with a probability *P* and rewiring them to the local neighbourhood [34].

**Figure 3.**
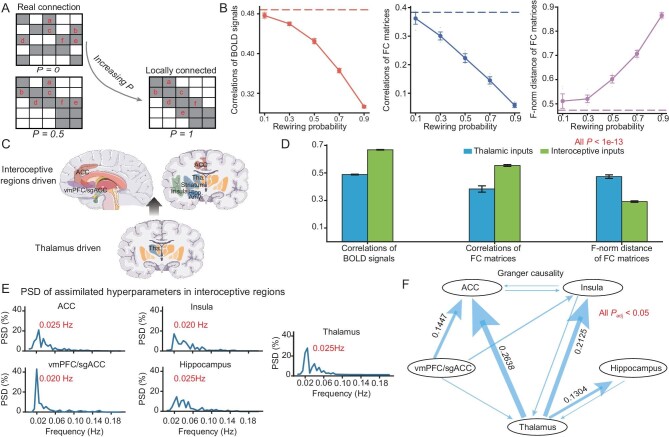
Investigating neuroanatomical architecture of the resting state and exploring the resting states driven by interoceptive regions. (A) Schematic of the rewiring procedure. When the rewiring probability *P* approaches 0, the network is close to the actual biological DWI structure. As *P* increases, the network connections become primarily locally connected. (B) Performance of the model in resting state with respect to rewiring probability *P*. Ten repetitions of simulation experiments were conducted on models with an average synaptic connection in-degree of 100, network sizes of 200 million and rewiring probabilities of 0.1, 0.3, 0.5, 0.7 and 0.9. The dashed line represents simulated results under the actual DWI structure (*P* = 0). The evaluation metrics are consistent with those in Fig. 2D. (C) Schematic representation of resting-state digital experiments driven by inputs from interoceptive regions, encompassing the hippocampus, insula, anterior cingulate cortex, vmPFC/sgACC and all subcortical regions. (D) Comparison of resting states driven by interoceptive subcortices and those driven by thalamus using the same three evaluation metrics as illustrated in Fig. 2D. (E) Power spectral density analysis of assimilated hyperparameters in interoceptive regions. The assimilated hyperparameters of input currents obtained from the interoceptive brain regions were averaged across five regions: hippocampus, insula, anterior cingulate cortex, vmPFC/sgACC and thalamus. (F) Time-domain conditional Granger causality analysis results for the assimilated hyperparameters of input currents derived from interoceptive brain regions across the same five regions. *P*-values were adjusted using Bonferroni correction.

Specifically, by varying the value of *P* from 0 to 1, the artificial voxel-wise architecture gradually varies from the original DWI-data-based architecture to a k-nearest neighbour graph (Fig. 3A). For each artificial voxel-wise architecture generated with rewiring probability *P*, we employ the same approach to sample the neuron-to-neuron network, as mentioned in the Supplementary Data. The simulated BOLD signals of these networks (with different rewiring probabilities *P*) are driven by inputs with the same assimilated hyperparameters of the voxels of the interoceptive or perceptive ‘input’ regions: the thalamus for the resting states. Both the correlations in voxel-wise BOLD time courses and the correlation in the region-wise FC matrices between the simulated model and biological data decrease with *P*, whereas the F-norm distance of the FC matrices between the simulated model and biological data increases with *P* (Fig. 3B). Hence, in the resting-state scenario, this rewiring destroys the similarities between the model and the real brain, which explicitly indicates the role of DWI-based architecture.

### Exploring the resting states driven by the interoceptive subcortex

The afferent/ascending neural pathways and neural circuits of interoception are known to convey information on the internal state of the body [35]. We further investigate the influence of interoceptive circuits on the resting state by using them as the ‘input’ regions, whose resting-state BOLD signals are assimilated. Herein, we consider five regions: hippocampus, insula, anterior cingulate cortex, vmPFC (ventromedial prefrontal cortex)/sgACC (subgenual anterior cingulate cortex) and thalamus as the interoceptive ‘input’ regions to be fitted for their voxel-wise BOLD signals (Fig. 3C). When simulating the model with 1 billion neurons and an average in-degree of 100, the similarities between the simulated signals and the biological BOLD signals of the whole brain are significantly larger than when using only the thalamus as the ‘input’ region. The mean PCCs of the voxel-wise BOLD time courses between the simulation and biological data are 0.668 and 0.488 when taking the interoceptive circuits and the solely thalamus as the ‘input’ regions respectively; the PCCs of the FC matrices are 0.554 and 0.383 accordingly. It is evident that incorporating the interoceptive region as the input has significantly enhanced the resemblance between the assimilated resting-state BOLD signals and the biological data. By employing a two-sample t-test, we observe a substantial improvement in the similarity between the assimilated resting-state BOLD signals and the biological data when incorporating the interoceptive region as input. Specifically, the t-values are as follows: t = 158.1 for the mean PCC of time courses, t = 21.3 for the PCC of FC matrices and t = −38.3 for the F-norm distance of FC matrices, all with *P*-values <1e-13 (Fig. 3D).

The mean activity was calculated by averaging the time series of assimilated hyperparameters of input currents over voxels from each interoceptive region: hippocampus, insula, anterior cingulate cortex, mPFC (medial prefrontal cortex)/sgACC and thalamus. Power spectral density (PSD) analysis has revealed that the highest spectral peaks of these five regions are at 0.02–0.025 Hz, and several lower peaks are ∼0.02–0.08 Hz (Fig. 3E).

Subsequently, we subject the averaged current hyperparameter sequence to conditional Granger causality analysis. We observe relatively strong causal relationships from the thalamus to the ACC (anterior cingulate cortex), from the thalamus to the insula, from vmPFC/sgACC to the ACC, and from the thalamus to the hippocampus (Fig. 3F). Moreover, most of the significant causality links can be statistically validated by other approaches, for instance, partial cross mapping (PCM) [36] (Fig. S12). The frequency domain Granger causality further shows that this driving effect of the thalamus primarily works on frequencies around 0.02 Hz (Fig. S8).

### Effect of scaling and architectural destruction during tasks

**Figure 4.**
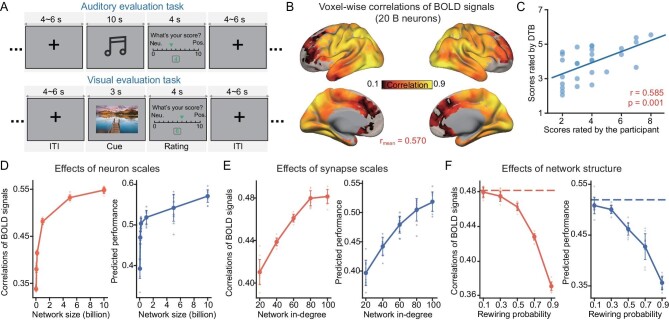
Scaling analysis and neuroanatomical architectural investigation of the cortico-subcortical model during tasks. (A) Schematic representation of the auditory and visual evaluation task. (B) Voxel-level PCCs between the assimilated, task-performing digital brain consisting of 20 billion neurons and the biological brain. (C) Prediction performance of the assimilated digital brain performing the auditory evaluation task. (D–F) Performance of the model concerning the number of neurons in the network, the average synaptic connection in-degree and the rewiring probability *P*. The evaluation metrics are the same as those illustrated in Fig. 4B and C. Ten repetitions of simulation experiments were conducted, consistent with the model used for resting-state experiments.

These phenomena, due to the influence of the neuronal and synaptic scales on the similarity between the assimilated model and its biological counterpart, can be observed from the BOLD signals during tasks. We consider the auditory evaluation task for the assimilated cortico-subcortical model (with different synaptic scales) and compare it with the biological counterpart: the SUBJECT's BOLD signals and evaluation scores. The assimilated model is established by fitting the BOLD signals of the voxels of the perceptive ‘input’ region, the primary auditory cortex (A1), by estimating the hyperparameter of the Gamma distribution of the input current received by neurons in this region, similar to what was done in ref. [25], with 20 billion neurons and an average in-degree of 100 (Fig. 1G and H, and Fig. 4A). The voxel-wise mean PCC over all voxels between the assimilated and biological BOLD signals is 0.570 (Fig. 4B) and the correlation between the predicted and the experimental evaluation scores is significant (r = 0.585, *P*-value = 0.001) (Fig. 4C).

When simulating the BOLD signals of the neuronal network models of different numbers of neurons or average in-degrees, in the same way as mentioned above, the similarities between the simulated BOLD signals and the biological counterpart increase with both the number of neurons and average synaptic connection in-degree. The mean PCCs between the voxel-wise time courses of the simulated and biological BOLD signals increase with the number of neurons, if the average in-degree is 100 (Fig. 4D left panel), and with the average in-degree, if the number of neurons is 1 billion (Fig. 4E left panel).

The influences of synaptic scale were also confirmed by the PCCs between the evaluation scores predicted by the simulated BOLD signals of the cortical and subcortical models, using the same linear regression model obtained from the biological data, and the real scores of the biological counterpart. These mean PCCs between the predicted and real scores increase with the number of neurons, if the in-degree is 100 (Fig. 4D right panel), and with the average in-degree, if the number of neurons is 1 billion (Fig. 4E right panel).

We also carry out the same architectural destruction through rewiring for the auditory evaluation tasks, as was done to the resting-state model. Both the correlations of the voxel-wise BOLD time courses and the correlation of the evaluation scores between the model predictions and the biological counterpart increase with *P*, too (Fig. 4F). This implies that the destruction of DWI-based architecture on the neuronal network model has the same influence on the task performance as the resting-state.

These results were also replicable for the visual evaluation task. By the same analyses and metrics, one can also observe that the similarities between the simulated models and the biological counterpart increase with the number of neurons and average in-degree in the computational cortico-subcortical models (Fig. 4A and B and Fig. S4).

### Digital lesioning of visual pathways

**Figure 5.**
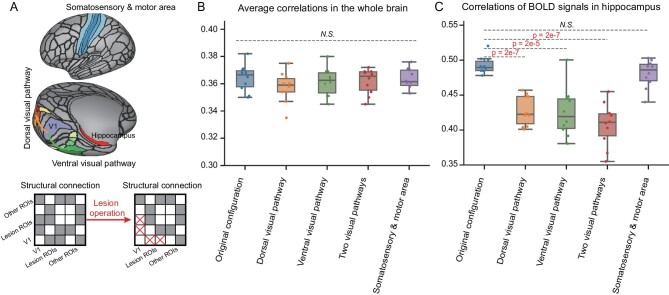
Digital structural lesioning operation on visual pathways impacted the neural dynamics of the hippocampus in the cortico-subcortical model in the visual evaluation task. (A) Schematic of the location of the primary visual cortex (i.e. V1), the visual pathways (the ventral visual pathway at the bottom and the dorsal visual pathway at the left), the hippocampus (i.e. the target region) and somatosensory and motor area (i.e. brain regions unrelated to vision) (upper), as well as an illustration of the lesioning operation of the structural connections (lower). (B) Comparison of the correlations between BOLD signals generated by the model and those of its biological counterpart, with different lesioning operations. (C) Comparison of correlations between hippocampal BOLD signals generated by the model with different lesioning operations, and those of its biological counterpart.

The cortical and subcortical computational models offer a possible framework for conducting certain ‘digital lesioning’ operations to the ‘neuroanatomical’ architecture, and for observing their influence on cognitive functions, which is something that cannot be done with a real subject. Herein, we demonstrate this possibility by conducting removal operations of the synaptic connections from the primary visual region (V1) to the basic visual pathways: the dorsal and ventral visual pathways (Fig. 5A). We have found a significant influence of these operations on the memory region, that is, the hippocampus; in comparison, however, the influence on the whole brain is limited.

To illustrate this, we calculate the similarities of the resting-state BOLD signals between the simulated model and the biological counterpart under the scenarios of digital lesioning to the visual pathways, in comparison to the original architecture. The mean PCCs between the BOLD time courses of the voxels at the hippocampus as generated by the simulated model, and those from the biological subject, significantly decrease from 0.493 to 0.428 when removing the connections from V1 to the dorsal pathway, from 0.493 to 0.425 when removing the connection from V1 to the ventral pathway, and from 0.493 to 0.408 when removing the connections from V1 to both the dorsal and ventral pathways. In comparison, when removing the connections from V1 to the motor areas, the mean correlation at the hippocampus only changes slightly (Fig. 5C).

The severing of connections from V1 to these regions does not influence the similarity between the simulated model and its biological counterpart in cortical and subcortical regions (Fig. 5B). This has validated, in a digital way, the specific role of the visual pathway on the hippocampus that has a major role in memory and learning.

## DISCUSSION

It has been widely acknowledged that the simulation of the human brain at the whole-brain scale and assimilation with biological structural and functional data is very difficult, and demands both the power of supercomputing and complicated methods for statistical inference [37]. However, considering the cost it demands, whether and how the reconstruction and simulation of the human brain could bring about benefits for understanding the brain's cognitive and intelligent functions has been vague in terms of quantitative methods. In this article, with the developed reverse engineering approach in the DTB [11,25], we have presented how the similarities between the assimilated cortico-subcortical models and the biological counterpart are affected by the neuronal and synaptic scaling, in terms of the number of neurons, the average synaptic connection in-degree and the neuroanatomical architecture. Generally speaking, we have found that the more similar the brain model is to the biological counterpart in both scale and architecture, the more similarity we have between the simulated model and the real brain both in resting state and during tasks, with solid quantitative metrics for the first time, despite the existence of a few platforms of neuronal network simulation [7,8, 12].

The computational neuronal network of cortex and subcortex offers a platform for discovering complex relations between brain architectures and corresponding high cognitive functions. The central limit theory may partially explain the correlation between the estimated time course and its noisy simulation. However, the influence of model scales on the performance of the computational model in terms of resembling its biological counterpart cannot be totally interpreted only by the central limit theory, because the similarity metrics also showed going monotonically to saturation asymptotically in the specific brain regions that have not been estimated, as the model scale increases. The interoceptive and perceptive schemes combined with the model architecture based on the structural data may partially account for these observations. Furthermore, the avalanche analyses show that the scaling of the model is crucial for allowing the brain to stay close to the critical state. When the number of cortical and subcortical neurons is as large as 1 billion, similar to the scale of a cat's brain [10], the simulated model starts to show a power-law characteristic.

This framework also presents a DTB platform for digitally trying and testing diverse cognitive and medical approaches that cannot otherwise be carried out on a real biological subject. The hypothesis that interoception is implicated in the informatic relation between the brain's resting states and the body can be tested through the assimilation approach by showing the interoceptive circuit's capability to enhance the similarity between the computational model and the biological data, in contrast to using advanced regions (left dorsolateral prefrontal cortex) as input (Fig. S11). The spectral analysis has shown that the main spectral peaks and the derived causality from the thalamus lie around 0.03 Hz for respiration [38,39] and 0.04 Hz for cardiac activity [39,40]. Furthermore, the digital operation provides a way to test hypotheses about the roles of brain regions/circuits/lesions in specific functions, in terms of dynamical mechanism, which cannot be conducted in a biological subject. This undoubtedly extends the scope of computational methods and supports the rationality of developing the DTB for neuroscience and brain health.

We argue that the present framework distinguishes itself from similar existing works in many aspects. First, the spiking neuronal network of the whole-brain scale, with up to 20 billion neurons and data-constrained structures, has been established, which is unique in terms of scale and multimodal structural constraints at the voxel level. Second, the data assimilation approach demonstrates its effectiveness in estimating the ‘large’ model by adeptly fitting BOLD signals in both resting state and action. Our data assimilation approach introduces a unique paradigm for assimilating tasks, which is an important aspect that has not yet been extensively explored in other methods or projects.

There are some limitations to this research. Firstly, we only have limited biological data for constructing the model. We have shown that the influence of the similarity between the model and the brain is essential to imitating brain dynamics and functions. The low spatiotemporal resolution of imaging data may result in impreciseness. In the future, integrated with other experimental data, including MEG/EEG data as well as detailed structural knowledge for specific brain regions or circuits, the reliability of our approach may be increased at a finer temporal scale. Second, computing capability limits prevent us from simulating the cortex and subcortex on the same synaptic scale as the human brain, which is believed to be on average as large as 10 000 synapses per neuron in the cortex [41]. However, our results have partially demonstrated that the model equipped with a larger in-degree is promising for better performance in terms of functional similarity. Third, one of the major characteristics of the pipeline in this work is the individual model based on personalized biophysical data, in comparison with existing works based on the ‘average’ brain over a group of subjects. We highlight that it is of significance to utilize individual biological neuroimaging data to construct personalized digital brain models, especially for investigating individual responses to specific tasks and individual-specific pathological characteristics. More importantly, this framework for constructing an individual digital brain model can be easily generalized to many other subjects.

In summary, understanding the relationship between brain structures, dynamics and cognitive/intelligent functions is essential for developing efficient diagnoses and treatments of brain diseases and more importantly, novel theories and technologies of brain-inspired intelligence. Making a digital version of the real brain, i.e. the DTB, is an efficient approach with regard to this aim, and represents a serious challenge to new computation hardware and systems, as well as computing methods for reverse engineering the brain, and this should be our research focus in the long term.

## MATERIALS AND METHODS

### Biological data acquisition and preprocessing

We scanned multimodal MRI from a single subject, as was done in ref. [25], via a 3 Tesla MR scanner. A high-resolution T1-weighted (T1w) image acquired with a rapid gradient echo sequence, as well as multi-shell DWI and fMRI data acquired with gradient echo-planar imaging (EPI) sequences, were obtained to extract the VBM of gray matter, structural connectivity and BOLD signals, respectively. After preprocessing, a series of data-cleaning procedures were implemented to integrate multimodal neuroimaging data into our DTB model more effectively, resulting in a cortico-subcortical model with a total of 16 043 voxels.

### Cortico-subcortical model

We applied a similar approach to the DTB model [25] with necessary modifications. Specifically, we used the same LIF neuron with AMPA and GABA synapses. The model architecture comprised 374 areas of the cerebral cortex and the subcortex in the parcellation of HCPex [42]. The coupling of the network was constrained by long-range neural fibre tracts as identified with DWI-based tractography and synaptic density given by SV2A PET [28]. Each voxel, containing excitatory and inhibitory neurons (roughly 4 : 1), was modelled as a micro-column (cerebral cortex, eight populations) [26,27] or a simple randomly connected circuit (subcortex, two populations). In this way, for a preassigned number of neurons and average synaptic connection in-degree, one could sample a neuronal network with neuron-to-neuron connections.

### Statistically inferring the cortico-subcortical model

We implemented the simulation of the neuronal networks by the graphics processing unit (GPU) clusters via several technologies to boost their performance [11]. We utilized the Vw-dHMDA method that integrates the hierarchical mesoscale data assimilation (HMDA) method [24] and the diffusion skill [43] to fit the voxel-wise experimental BOLD signals. The performance of the data assimilation was measured by the following metrics: (i) PCCs between the time courses of simulated BOLD signals generated by the neuronal network and the Balloon-Windkessel model [30], and those from its experimental counterpart; (ii) PCCs between the FC matrices obtained by the simulated model and the experimental data; (iii) the F-norm of these two FC matrices. We sampled the neuronal network structure with different numbers of neurons and average synaptic connection in-degrees to explore the influence of the neuronal and synaptic scales.

### Assimilating brain dynamics in resting states and during tasks

We utilized the Vw-dHMDA to separate sets of voxels in specific brain regions as the ‘input’ regions and then simulated the cortico-subcortical models. For the resting-state scenario, we considered some subcortical regions: the thalamus served as the relay spot [44]. We also exploratively considered interoceptive regions [45] as the inputs for the resting states. Avalanche criticality and phase synchrony analyses were performed [23].

For the auditory and visual tasks, we considered the primary auditory cortex (A1) and the primary visual cortex (V1) as the inputs respectively. We utilized the simulated BOLD signals to predict task scores by the regression model trained with the experimental data, following ref. [25].

### Digital brain structural destruction

The computational model provided a way to carry out digital operations on brain structures. We considered two destruction scenarios for evaluating the extent to which the resting-state dynamics and task performance could depend on the underlying network connectivity. This first method systematically destroyed network topology by randomly rewiring edges to local neighbourhood nodes while preserving network size and degree distribution [34]. The second assessed the prediction performance of tasks by a digital lesioning operation by removing connections from V1 to the dorsal and ventral visual pathways respectively [46]. We then simulated the neuronal network after the digital operation and compared its BOLD signals to the original ones.

## Supplementary Material

nwae080_Supplemental_File
