# Supplementary material for: Imitating and exploring the human brain's resting and task-performing states via brain computing: scaling and architecture
Source: Natl Sci Rev. 2024 Mar 1;11(5):nwae080. doi: 10.1093/nsr/nwae080 (PMC11129584; doi:10.1093/nsr/nwae080)
Supplement: nwae080_Supplemental_File [file nwae080_supplemental_file.pdf]

Supplementary Data

Supplementary Methods and Materials

Computational cortex and subcortex model

The model comprises 374 areas of the brain network in the parcellation, henceforth referred to as HCPex[1]. Each area contains some voxels and each of them is modelled as a micro-column structure for the cerebral cortex or a random sub-network for the subcortex. We here give a brief overview of the construction principles underlying the network definition and refer to our previous paper [2] for details of the derivation and an analysis of the network structure. Table 1 summarizes the construction principles leading to the population sizes and the area-, layer-, and population-specific connectivity map.

Within every voxel, distinct populations of excitatory (exc.) and inhibitory (inh.) neurons are present across the cortical layers L2/3, L4, L5, and L6 [3,4]. Notably, layer 1 is devoid of neuronal presence in our consideration. To be consistent with previous studies, only voxels distributed in the cortex are considered to be characterized as a micro-column (8 populations), and other voxels located in the subcortical region are represented by a randomly connected subnetwork involving two populations (ext. and inh.). Population sizes in the model are derived from a collection of data on laminar and total cortical thicknesses, voxel-based morphometry (VBM) of gray matter, and a predefined total simulation scale S (default is 1 billion). The ratio between excitatory and inhibitory cells is layer-specific and roughly 4:1 on average.

Based on the experimental finding, we assume the in-degree for each voxel (equally says synaptic density, because the same volume for each voxel) is proportional to the SV2A PET[5] and related to the predefined in-degree of V1 (also abbreviated as average in-degree without ambiguity and denote as d, default is 100), which leads to larger numbers of synapses onto neurons in higher areas. We distinguish synapses in a neuron into 2 distinct types. The first is some internal synapses originating within each micro-

column (local connection), and the second is cortico-cortical synapses formed by projections from other voxels (long-range connection). Only excitatory synapses are allowed for long-range connections in our model. We adopt the population-specific connectivity matrix (Table 2) to refine the structure for cortical voxels, meanwhile keeping the average number of incoming synapses for the corresponding voxel constant. The components for each population in the last column of (Table 2, cc) determine the ratio between the numbers of local synapses and cortico-cortical synapses. The coupling among voxels is constrained to long-range neural fiber tracts as identified with diffusion-weighed imaging (DWI)-based tractography. Specifically, For each population (destination), we compute the total number of incoming synapses of long-range connection and then distribute synapses to each excitatory population (source) according to the normalized DWI and corresponding relative layer depth. Overall, long-range inputs amount to approximately 35% of the total inputs to each neuron in the network according to the inner-outer ratio as in Table 2. These derivations lead to a connectivity map with similar but non-identical local circuits. The connectivity between voxels shows high heterogeneity and sparsity with the density of non-zero connections at 1.6%.

The resulting high-resolution neuronal network can be sampled with different predefined scales S and in-degrees d. We still use the leaky integrate-and-fire neuron with AMPA, GABA (denote as type u) synapses as the basic soma and synaptic connection units as[2]:

$$\begin{aligned} C_i\dot{V}_i &= -g_{L,i}(V_i - V_L) + \sum_u g_{u,i} J_{u,i}(V_u - V_i) + I_{bg,i} + I_{ext,i} \\ \dot{J}_{u,i} &= -\frac{J_{u,i}}{\tau_i^u} + \sum_{k,j} w_{ij}^u \delta\left(t - t_k^j\right) \end{aligned} \quad (1)$$

While the number of synapses is population-, layer- and area-specific, we make a simple choice for the synaptic weights to restrict complexity: Individual weights w_{ij}^u are drawn from uniform distribution $U(0,1)$. For neurons in the excitatory population, the external current $I_{ext,i}$ mimics the input from the environment and distributes with a Gamma distribution with a shape parameter α=5 and inverse scaling parameter $\beta =$

$5 / I_{ext,i}^{\mathcal{H}}$ for type u synapses in i population. The hyperparameter$I_{ext,i}^{\mathcal{H}}$ is learned from the experimental BOLD signal through data assimilation (DA) in different task setting. We choose single-cell parameter values equal to those used[6] (cf. Table 3). By using a simple neuron model, we limit the complexity of the simulations to bring out the influence of the network connectivity.

Table 1: Model description.

Model summary	
Brain part	2 brain parts consist of: the cortex and sub-cortex
Topology	Voxel-wise DWI is normalized (row normalization) as the connection probability.
Populations	The cortical voxel is described as a micro-column (8 populations for each) and the others are modeled in a random-connected manner (Exc. and Inh. 2 populations).
Connectivity	Population-specific but otherwise random.
Neuron model	Leaky integrate-and-fire (LIF).
Synapse model	Conductance-based chemical synapse (2 types), exponential postsynaptic currents. AMPA type is modeled in excitatory projection; GABA type is modeled in inhibitory projection.
Background input	OU-type external current.
Neuronal size	We assign the number of neurons in each voxel is proportional to their corresponding VBM, laminar, and total thickness. the ratio of the number of excitation neurons over that of inhibitory neurons of each layer equals 4:1.
**Input**	
OU current	The background current is given by independent Ornstein Uhlenbeck (OU) processes and described as: $$\tau_{bg} dI_{bg,i} = (\mu_{bg} - I_{bg,i})\mathrm{dt} + \sqrt{2\tau_{bg}}\sigma_{bg} dW_t.$$ The parameters are the same among neurons ($\mu_{bg} = 0.4\ \mathrm{nA}, \sigma_{bg} = 0.15\ \mathrm{nA}, \tau_{bg} = 4\ \mathrm{ms}$).
**Connectivity**	
Type	The source and target neurons are drawn randomly without replacement (not allowing autapses and multapses) with population-specific connection probabilities.
Population-specific connection probability.	The count of inner and outer synaptic edges of each population is decided by the DWI and micro-circuit (micro-column or voxel structure). The detailed micro-circuit inside the voxel of the cortex and subcortex is listed in Table 2.
in-degree	The related average in-degree of neurons in a voxel of V1 equals to a constant. We set $d = 100$ for default.
Weight	fixed, drawn from uniform distribution $U[0, 1]$.

Table 2: The detailed microcircuit in our model essentially represents two different cases: Cortex and Subcortex. The micro-column distributed in the cortex is organized as a laminar model, and the others in the subcortex are characterized as a randomly connected subnetwork. Each row represents a postsynaptic neuron type, where e1 (i1) indicates the excitatory (inhibitory) neuron of layer 1, and so on. Each column represents a presynaptic neuron type, where CC indicates the presynaptic neuron is in other cortex areas, respectively.

Cortex											
		presynaptic									
		i1	e2/3	i2/3	e4	i4	e5	i5	e6	i6	CC
postsynaptic	e1	1323	823	200	15	1	9				10133
	i1	901	560	149	10	1	6				6899
	e2/3	133	3554	804	881	45	431		136		1020
	i2/3	52	1778	532	456	29	217		69		396
	e4	27	417	84	1070	782	79	8	1686		1489
	i4		168	41	628	538	36		1028		790
	e5	147	2550	176	765	99	621	596	363	7	1591
	i5		1357	76	380	32	375	403	129		214
	e6	2	643	46	549	196	327	126	925	597	2609
	i6		80	8	92	3	159	11	76	499	1794

Subcortex				
		presynaptic		
		e	i	CC
postsynaptic	e	0.56	0.2	0.24
	i	0.56	0.2	0.24

Table 3: Default parameters in our model in numerical simulation.

Neurons		Synapse		OU input	
C	0.5 nF	V_{ampa}	0 mV	μ_{bg}	0.4 nA
g_L	25 nS	V_{gaba}	-70 mV	σ_{bg}	0.15 nA
V_L	-70 mV	τ_{ampa}	2 ms	τ_{bg}	4 ms
V_{thr}	-50 mV	τ_{gaba}	20 ms		
V_{reset}	-55 mV	w_{ij}^{u}	$U[0, 1]$		
T_{ref}	2 ms				
g_{ampa}	2 nS				
g_{gaba}	10 nS				

Hardware and Software Environment for simulation

The C++ program using C++ compiler (g++) version 7.3.1, was developed with MPI library functions and rocm-4.1.0 hipcc compiler for GPU. The Python program for data assimilation was developed using Python version 3.7.11. The Python and C program runs in a Linux environment.

The cluster in Advanced Computing East China Sub-Center has a total of 3503 computing nodes, each of which has single 32-core processors operating at 2 GHz and 128 GB of DRAM memory. Each node also has 4 GPUs operating at 1.10 GHz and each GPU has 16GB HBM2 working on 800MHz, with 1TB/s memory bandwidth. GPU communication between each other within one node is through the share memory, while communication across nodes is through a 200Gbps Full Duplex Infiniband network.

Data acquisition and preprocessing

In this work, we scanned multimodal MRI from the corresponding author of this paper, Jianfeng Feng. All neuroimaging was performed on a 3 Tesla MR scanner (Siemens Magnetom Prisma, Erlangen, Germany) at the Zhangjiang International Brain Imaging Centre in Shanghai, using a 64-channel head array coil. High-resolution T1-weighted (T1w) images were acquired using a 3D Magnetization-Prepared RApid Gradient Echo (3D-MPRAGE) sequence (TR/TE = 3000/2.5 ms, TI = 1100 ms, flip angle = 7°, FOV: 320*320*240, Voxel size: 0.8*0.8*0.8 mm3). Multi-shelled diffusion-weighted images (DWI) were acquired using a single-shot spin-echo planar imaging (EPI) sequence (TR = 3200 ms, TE = 82 ms, FOV: 140*140*92, Voxel size: 1.5*1.5*1.5 mm3, multiband factor = 4, phase encoding: anterior to posterior) with two b-values of 1500 s/mm2 (30 diffusion directions) and 3000 s/mm2 (60 diffusion directions), in which B0 images were interleaved in every 6 volumes. B0 images with the same DWI protocol using an opposite polarity (i.e., phase encoding from posterior to anterior) were also acquired. fMRI data were acquired using a gradient echo-planar imaging (EPI)

sequence (TR = 800 ms, TE = 37 ms, flip angle = 52°, FOV: 104*104*72, Voxel size: 2*2*2 mm3, multiband factor = 8, phase encoding: anterior to posterior). The resting-state fMRI scan consisted of 400 contiguous EPI volumes and task-based fMRI scans consisted of 350 and 570 EPI volumes in visual and auditory evaluation tasks, respectively.

The voxel-based morphometry (VBM) of T1w images was preprocessed by the VBM8 toolbox in the Statistical Parametric Mapping package (SPM, http://www.fil.ion.ucl.ac.uk/spm). Briefly, the gray matter image was segmented and normalized to Montreal Neurological Institute (MNI) space by a nonlinear registration. Finally, the normalized image was smoothed with a full-width at half-maximum (FWHM) 8-mm Gaussian kernel and resampled at a resolution of 3*3*3 mm3.

FSL software V6.0.4 (Functional Magnetic Resonance Imaging of the Brain Software Library, http://www.fmrib.ox.uk/fsl) and MRtrix 3.0 (http://www.mrtrix.org) 1 were utilized to preprocess DWI data. The DWI was denoised and corrected for Gibbs ringing artifacts 2-5. The DWI was then corrected for head motion, eddy current, and tissue susceptibility-induced off-resonance geometric distortions with the reversed phase encoding b = 0 s/mm2 images 6-8. The DWI bias field created by the nonuniform coil receives sensitivity was also corrected. For the anatomical connectivity matrix, we first generated a mask image appropriate for seeding streamlines on the gray matter-white matter interface. White matter tractography was used to estimate the fiber tract numbers connecting each pair of voxels in the mask image, resulting in an anatomical connectivity matrix. The connection of a voxel to itself was set to 0 in the connectivity matrix for the simulations.

For BOLD signals, the following preprocessing was performed using fMRIPrep 9. First, a reference volume and its skull-stripped version were generated using a custom methodology of fMRIPrep. A deformation field to correct for susceptibility distortions was estimated based on fMRIPrep's fieldmap-less approach. The deformation field is that resulting from co-registering the BOLD reference to the same-subject T1w-reference with its intensity inverted. Registration is performed with antsRegistration

(ANTs 2.3.3), and the process regularized by constraining deformation to be nonzero only along the phase-encoding direction, and modulated with an average fieldmap template. Based on the estimated susceptibility distortion, a corrected EPI (echo-planar imaging) reference was calculated for a more accurate co-registration with the anatomical reference. The BOLD reference was then co-registered to the T1w reference using flirt with the boundary-based registration cost-function 10,11. Co-registration was configured with nine degrees of freedom to account for distortions remaining in the BOLD reference. For resting-state data, ICA-based automatic removal of motion artifacts (AROMA) was used to generate aggressive noise regressors as well as to create a variant of data that is non-aggressively denoised 12. Then the preprocessed data were smoothed with a full width at FWHM Gaussian kernel of 6 mm and filtered with a band-pass filtering (0.01 ~ 0.1 Hz). Finally, the preprocessed data were resampled at a resolution of 3*3*3mm3.

To integrate multimodal neuroimaging data into our neural network model more effectively, we applied a series of data cleaning procedures based on the following principles: (1) For cortical structures, including the cerebellum, only voxels with a gray matter volume (GMV) greater than 0.4 were retained. (2) For subcortical structures with a GMV greater than 0.2 were kept. (3) Isolated voxels, which do not form structural connections with any other voxel, were removed. Hence, a total of 16,043 voxels (cortex: 14,473, subcortex: 1,570) were included in the following model construction.

Voxel-wise diffusion hierarchical mesoscale data assimilation (Vw-dHMDA)

In this work, we employed the diffusion ensemble Kalman filter[7] and the hierarchical mesoscopic data assimilation (HMDA) [8] to estimate the hyperparameter of the external currents of neuron for each voxel by fitting the simulated BOLD signals to experimental BOLD signals, where the simulated BOLD signals are generated using the Balloon-Windkessel model[9].

Specifically, we take each voxel (both micro-column in cortex and 2-population

subnetwork) as the ROI (region of interest) and assume that excitatory neurons with the same type share a common distribution for the parameters (e.g. the external currents), or equivalently, the same set of hyperparameters $I_{ext,i}^{\mathcal{H}}$. In practical implementation, parameter inference is executed using the diffusion ensemble Kalman filter, while hyperparameter inference employs a straightforward random-walk bootstrap filter[8]. The distributed Kalman filter provides an effective approach to managing high-dimensional observations, effectively mitigating computational complexity and addressing the ill-posed nature stemming from the constrained number of data time points.

Consequently, we employed Vw-dHMDA[2] for the fMRI data over the neuronal network and hemodynamic models by taking all states of neuronal including neural activities (spikes), synaptic currents, variables of hemodynamics, bold signals, and the parameters to be estimated as well as its preassigned distribution with the hyperparameters. Analysis and prediction of the Vw-dHMDA filter is executed at each time point of the BOLD signals. The time scale follows the biological clock (in ms) and takes each time step as the period of the fMRI scanning. After estimating the hyperparameter of a subnetwork with an observation signal (experimental BOLD signal), we re-simulate this model by assigning the external current for each neuron according to the hyperparameter series $I_{ext,i}^{\mathcal{H}}$ and then evaluate the fitting effect.

In practical implementation, the hyperparameters of the external currents to the neurons of the voxels in the input brain region are estimated by the Vw-dHMDA algorithm in the cortex and sub-cortex model of DTB of 200 million neurons and average in-degree as 100. The network structure is modeled and sampled from Jianfeng's sMRI and DWI data incorporated with the PET data, as mentioned in Section 1. We utilized separate sets of voxels in specific brain regions in the HCPex template nodes to simulate various samples of brain models. Subsequently, we gathered population-level spike rates from each voxel (ROI) and then compute the voxel-level hemodynamics as the BOLD signals. We conducted iterations of the Vw-dHMDA

process to adjust the hyperparameters and then proceeded to resample and update neuron parameters based on the hyperparameters on each simulation node. By this approach, we successfully achieved the assimilation process to align the BOLD data with the computational neuronal network model.

Evaluation metrics

We utilize several metrics to evaluate the performance of the assimilated computational model with the biological data of the counterpart. To validate the assimilated model in the resting states, we assessed the agreement between its spatiotemporal patterns and those observed in experimental data. First of all, we used the Pearson correlation coefficients between the simulated BOLD time courses and the experimental fMRI data in voxels to measure the performance of the Vw-dHMDA. As done in [2], we alternative employ the following lag correlation coefficients:

$$pcc(x_{DTB}(t+lag), x_{exp}(t))$$

where $pcc(\cdot,\cdot)$ stands for the Pearson correlation coefficients, $x_{DTB,exp}$ for the time course acquired by the DTB and experimental counterpart and lag for the time delay. We take $lag = 3$ throughout the paper unless otherwise stated.

We also compared the functional connectivity (FC) networks acquired by the simulation DTB with the experiment counterpart. The FC is defined by the Pearson correlation coefficients between two BOLD signals $x_i(t)$ and $x_j(t)$ of two brain regions, i and j, respectively. Namely,

$$FC_{ij} = pcc(x_i(t), x_j(t))$$

In the voxel-wise fMRI, the BOLD signal of each brain region is obtained by simply averaging the BOLD signals over all voxels in this region. Herein, the difference between the FC networks of the simulation DTB, $\left[FC_{ij}^{DTB}\right]$ and the experimental counterpart $\left[FC_{ij}^{exp}\right]$ is measured by two metrics.

The first is the Pearson correlation coefficient between those two FC networks by

taking the matrices $[FC_{ij}^{DTB}]$ and $[FC_{ij}^{exp}]$ as two high-dimensional vectors, namely

$$pcc(vector([FC_{ij}^{DTB}]), vector([FC_{ij}^{exp}]))$$

Here, $vector(\cdot)$ stands for the operation that transform matrix into vector by a given order.

The other one is the Frobenius norm of the matrix difference between two FC networks, namely,

$$\|[FC_{ij}^{DTB}] - [FC_{ij}^{exp}]\|_F$$

Where $\|\cdot\|_F$ stands for the Frobenius norm.

Resting-state assimilation

To assimilate DTB in the resting states, we first conduct a grid search to initialize the conductance parameters of synapses $g_{ampa,i}$ and $g_{gaba,i}$, leading the neuronal network to an asynchronous irregular firing state. See [2] for more details. Each population in both the micro-column structure and voxel structure has identical conductance parameters.

In this work, we mainly consider the thalamus that serves as one of the main relay spot for the majority of sensory information[10]. Herein, the DTB approximates the input signal (external current I_{ext}) by tracking the empirical resting BOLD signal only from the thalamus and then simulates the signal's propagation within the best-fitted resting-state model. We employ the Vw-dHMDA on the thalamus to decode the sensory stimulus (it denotes the assimilated hyperparameters of the voxels in the thalamus). Then, we can simulate the cortex-subcortex model by sampling the values of external currents in the thalamus as mentioned before.

To more effectively replicate the mechanism where the resting-state of the brain is influenced by the body's internal sensory perception, we also consider other interoceptive processing brain regions, including hippocampus, insula, anterior cingulate cortex, vmPFC/sgACC, and all subcortical regions[11]. By the same way

mentioned above, we further assimilated the resting-state cortex and subcortex model by fitting the resting-state BOLD signals of the voxels in the interoceptive region.

To investigate the causal relationships among interoceptive regions, we implemented the conditional Granger causality analysis among the estimated singals in both time domain[12] and frequency domain[13]. The averaged input currents obtained from the interoceptive brain regions across five regions: hippocampus, insula, anterior cingulate cortex, vmPFC/sgACC, and thalamus are taken into analysis. The p-values were subjected to Bonferroni correction. The order of the model is determined to be 1 (0.8 seconds) based on the Bayesian Information Criterion (BIC). We also conducted frequency-domain analysis on the aforementioned current hyperparameters of the assimilated interoceptive brain regions after region averaging. The power spectral density (PSD) is estimated using Welch's overlapped segment averaging estimator.

Scaling experiment setting

The simulation of the whole DTB for the cortex and subcortex network from the assimilated model is implemented by sampling the parameter values of external currents $I_{ext,i}$ of the input brain regions for assimilating both the resting-state BOLD signals and the task BOLD signals from the assimilated hyperparameters accordingly and simulating the DTB. Then, we obtain the simulated voxel-wise BOLD signals for all cortex and subcortex for further analyses.

In this work, the assimilated hyperparameters can be employed to the DTB model with the same MRI-based structure but of different scales, including different numbers of neurons, and different average in-degree. Specifically, we constructed a number of networks with diverse scale of neurons: 50 million, 100 million, 200 million, 1 billion, 5 billion, and 10 billion, and in addition, established a number of networks with diverse average in-degree (d as mentioned above) from 20 to 100 with the default network size 1 billion, following the sMRI and DWI data from the same objective.

However, for the network with different average in-degree, the conductance parameters are simply scaled by:

$$g_{u,i,D} = g_{u,i,d}\frac{D}{d} \qquad (2)$$

Here, $g_{u,i,d}$ and $g_{u,i,D}$ stand for the conductance parameters (u=ampa, gaba) for the model with degrees d and D respectively. Simulating the DTB can be easily carried out by sampling the external current parameters from the distribution of estimated hyperparameters accordingly. The implementation of simulation is the same as mentioned before. Each trial is conducted by 10 repetition simulations of the DTB for each model to see the effects of randomness of the sampling processing.

Structure destruction experiment

In this work, we also simulate the DTB model with the same hyperparameters but via slightly modifying the network structure, in order to demonstrate how the dynamics and functions of the model are affected by the DWI-based network structure.

Specifically, we began from the original DWI connection, and randomly rewire the nonzero projection to a nearest neighborhood for each voxel with a given probability p. This method systematically destroys network topology while preserving network size, degree distribution, density, and the distribution of connection weights. With the probability p increase, the DWI topology is gradually evolving to a local-connected network. Importantly, this procedure was performed at the voxel resolution and the voxel sizes (i.e. the number of neurons) were not changed during the rewiring procedure. We applied the assimilated hyperparameters to models consisting of 1 billion neurons with an average in-degree of 100 and rewiring probability of 0.1, 0.3, 0.5, 0.7 and 0.9. Each trial is also conducted by 10 repetition simulations for powerful demonstration.

Power-law avalanche analysis

We conduct the criticality and phase transition analyses on the simulating data of the assimilated DTB and compared with their biological counterpart data. The details of these methods can be referred to (14).

Neural avalanches are identified from fMRI data as follows. First, a point process

is constructed from the standardized fMRI time series of each brain region by applying a threshold θ and by taking the peak of the fMRI signal within each suprathreshold time segment. Next, by binning these point processes with time windows of duration $\Delta t = 800$ ms (same as the fMRI time resolution), we obtain a spatiotemporal pattern of binary activation. A neural avalanche is then defined as a cluster of temporally contiguous active sites, that is, no active site before and after an avalanche[15,16].

Denote the duration T and size S (i.e., the total number of active sites) of each avalanche are then calculated. To characterize criticality, the avalanche duration and size are fitted with power laws as $p(T) \sim T^{-\tau}$ and $p(S) \sim S^{-\alpha}$, respectively. Similarly, the average avalanche size as a function of duration is fitted with $\langle S\rangle(T) \sim T^{\gamma}$. Theoretical studies suggest that for a system at criticality these exponents should satisfy $\gamma = \frac{\tau-1}{\alpha-1}$. Therefore, we define the quantity $\delta = \left|\gamma - \frac{\tau-1}{\alpha-1}\right|$ to measure how far away the system is from criticality[17] .

To investigate the critical dynamics of models of different scales, 120 repetitions of simulation were conducted on models with an average in-degree of 100 and network sizes of 0.05, 0.2, 1 and 5 billion. To assess the robustness of our findings, we conducted the avalanche criticality analysis mentioned above on the simulated BOLD signals using varying configuration parameters (thresholds and fitting windows for both avalanche size S and duration T, where θ in {2.3, 2.4, 2.5, 2.6, 2.7, 2.8}, $S_{min} \in [1,2]$, $S_{max} \in [10,38]$, $T_{min} \in [1,2]$, $T_{max} \in [10,22]$). We subsequently applied Clauset's goodness-of-fit test to assess the plausibility of these fits, which compared the Kolmogorov–Smirnov (KS) statistics in two scenarios: (1) between the data generated by simulation and the fitted model, and (2) between the data generated by power law models and the fitted[14,18-20]. We considered the data as fitting the truncated power law if the KS-statistic derived from the simulated data was less than the KS-statistic obtained from at least 10% of the power law models ($\rho \geq 0.1$) [14]. We calculated the ratio of the number of fittings that satisfy $\rho \geq 0.1$and the corresponding distribution of δ to all configuration parameters for models of different scales to evaluate the

criticality.

Phase transition analysis

We calculate the order parameter to investigate potential phase transitions in the model. In particular, we focus on the Kuramoto order parameter which characterize the phase synchrony of the system. We consider two sets of analyses, one for the phase synchrony between different voxels within each brain region and the other for the phase synchrony between different brain regions. These analyses are repeated for the resting state data of human brain and for digital brain simulations of different sizes (50m, 200m, 1b, 5b). Simulations are repeated for 10 trials, each with a duration of around 300 time steps (with an 800 ms time resolution). For human brain data, only a single trial is available, with the first 19 steps and the last 3 steps omitted from the analysis.

Let $x_j^\alpha(t)$ be the (post-processed) fMRI time series for voxel j inside a brain region α. To characterize the phase synchrony between voxels within a brain region, we apply Hilbert transform to $x_j^\alpha(t)$ for all $j \in \alpha$ to get the analytic signal

$$z_j^\alpha(t) = x_j^\alpha(t) + iH\left[x_j^\alpha\right](t).$$

The instantaneous phase of each voxel is then calculated as the principal argument of the analytic signal

$$\theta_j^\alpha(t) = \mathrm{Arg}\, z_j^\alpha(t)$$

The Kuramoto order parameter (KOP) is then calculated as

$$r^\alpha(t) = \left| \frac{1}{N_\alpha} \sum_{j=1}^{N_\alpha} e^{i\theta_j^\alpha(t)} \right|$$

Note the range of KOP is between 0 and 1, with 0 corresponding to asynchrony and 1 corresponding to perfect synchrony.

Finally, we calculate the mean, std, coefficient of variation, and entropy of each time series. We first calculate the average fMRI signal for each brain region

$$\bar{x}^\alpha(t) = \frac{1}{N_\alpha} \sum_{j=1}^{N_\alpha} x_j^\alpha(t)$$

This averaged signal is then used to calculate the KOP between brain regions $r(t)$ which is a scalar quantity describing the phase synchrony between brain regions inside the whole brain.

Task explorative assimilation

We take several digital explorative experiments to demonstrate the capability of emotion representation of the DTB, compared with the cognitive experimental data. The details of these digital experiments can be found in (2).

As a framework shown in Fig. 1G, first, according to the specific task, we assimilate the perceptive ROI by the task fMRI data in the whole cortex and sub-cortex neuronal network. We employ the Vw-dHMDA algorithm on this perceptive ROI (refer to “input” brain region) at the voxel level to decode the sensory stimulus. The simulation is redone by injecting the estimated currents into the model to virtually mimic the real-world experiment.

Secondly, the brain activation of the stimulus cue for each trial is first assessed with the BOLD signals from both the real and digital task brains by the general linear model, in which the regressors for modelling each trial is established by convolving the corresponding experimental condition with SPM’s canonical hemodynamic response function (HRF) and six head motion parameters are set as the additional covariate regressors. Hence, the patterns of brain activation are comparable between the digital brain and biological brain. We then train a linear regression model with the biological brain activations during the stimuli evaluation as the response variables and the real scores of the emotional pictures as the predictors. The least absolute shrinkage and selection operator (LASSO) regularization has been employed to pursuit a sparse coefficient vector. In this model, we remove the first two trails due to the bad quality of assimilation and exclude the brain activations in the input regions to minimize the confounding factors from the inputs. Finally, we predict how the subject rates real-world pictures with the brain activations from the digital brain and the sparse coefficient

vector obtained via LASSO.

Third, the performance of the assimilation of the given decision make tasks can be measured by several metrics. The first the Pearson's correlation coefficients between the assimilated BOLD signals and the corresponding real BOLD signals were computed as an estimation of the similarity between the digital task brain and the biological brain, as defined in (sec: Evaluation metrics). The second is the Pearson's correlation coefficients between the predicted evaluation scores by the DTB and the real scores recorded for the counterpart subject, aligned with the input sequence.

Auditory evaluation task assimilation

In the auditory evaluation task, the primary auditory cortex (A1) has been included as the perceptive input, which is responsible for the primary auditory information processing. Hence, we could obtain the assimilated signals of the whole brain and utilize them for score prediction. A total 15 trials were assessed in the auditory evaluation tasks. For each trial, after a 10-second sound cue, the participant was asked to evaluate how he felt to the real-world stimulus with a Likert scale from 0 to 10 in 4 seconds (ref. Fig. 4A).

Visual evaluation task assimilation

The visual evaluation task is assimilated in the exactly same way done on the auditory task. Specifically, the primary visual cortex (V1) is used to serve as the input region. A total 30 trials were assessed in the visual evaluation tasks. Each trial includes a 10-second sound cue and asking the participant to evaluate how he felt to the real-world stimulus with a Likert scale from 0 to 10 in 4 seconds (ref. Fig. 4A).

Explore effects of neuronal synapses scales

To investigate the impact of network size on the effectiveness of DTB in action, we simulate the assimilated cortex and sub-cortex model of DTB by sampling the injection current parameters by the same estimated values by the Vw-dHMDA, in the

neuronal network structures with different numbers of neurons as 50 million, 100 million, 200 million, 1 billion, 5 billion, and 10 billion respectively, given the average in-degree as 100 (ref. Fig. 4D, Fig. S5A).

To investigate the influence of synaptic connection in-degree on the performance of the auditory task of the DTB, we constructed the cortex and sub-cortex neuronal network model of different synaptic connection in-degree of 20, 40, 60, 80 and 100, respectively, given the number of neurons as 100 million. In the visual evaluation task, we fix the number of neurons of the DTB model as 10 billion and vary the synaptic connection in-degree as 20, 40, 60, 80 and 100. The remaining experimental procedures were consistent with those of the auditory evaluation task (Fig. 4E, Fig. S5B).

Each set of experiments above was repeated ten times for the network modelling and simulation. The means and stds of the results are plotted in Fig. 4D, Fig. 4E, Fig. S5A and Fig. S5B.

Digital operation to network structure

The DTB model presents a digital approach to operate the network structure and investigate their influence to brain dynamics and functions, which generally cannot be done on a biological subject. To demonstrate this capability, we design the following digital experiment of breaking the visual pathways and investigate their influence on the visual evaluation task as aforementioned, compared with the other pathways such as motor areas.

Specifically, after assimilating the input brain region (the primary visual region V1), we construct the neuronal networks of the cortex and sub-cortex DTB model by removing the synaptic connections from V1 to dorsal pathway (including IPS1、V3A、V3B、V6、V6A and V7) and from V1 to ventral pathway (including FFC、PIT、V8、VMV1、VMV2、VMV3 and VVC) respectively, beside the original network structure to compared with. In comparison, we also construct the networks by removing the synaptic connections from V1 to motor pathway (including 1、2、3a、3b and 4) and

from V1 to both the dorsal and ventral pathway respectively. All networks are of the same neuron numbers (1 billion) and the same average in-degree (100).

Then, we simulate the models above assigned with the assimilated hyperparameters to the external currents of the input V1 region. Herein, we focus the similarity of the BOLD signals of the hippocampus region between the simulated cortex and subcortex DTB models generated as mentioned above and the experimental data of the counterpart subject. We calculate the Pearson's correlation coefficients between the BOLD time course. Each DTB model with specific configuration is sampled and simulated for 10 times to obtain the mean the std of the correlation, as well as their distribution.

Supplementary Results

Supplementary experiments of in-degree investigation in resting-states

We have replicated the in-degree experiments still hold for a different number of neurons. Given the number of neurons 0.2 billion, it was shown in Fig. S1A that both the mean PCCs of the voxel-wise BOLD signals and the PCCs of the region-wise FC matrices between the assimilated model and the biological counterpart in resting-states increase, and the F-norm distance of the region-wise FC matrices between the model and the subject decrease with respect to the average in-degrees. All experiments were done following the same approaches and configuration as in the main text.

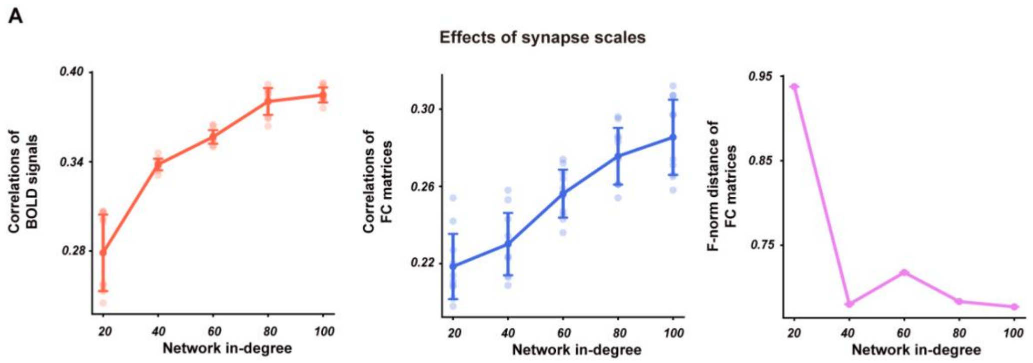

Fig. S1. Assessment of DTB performance in the resting state based on the average in-degree. Ten repetitions of simulation experiments were conducted on models with network sizes of 200 million and an average in-degree of 20, 40, 60, 80 and 100, using the same three evaluation metrics as shown in Figure 2D.

Supplementary illustration of avalanche criticality analyses

We have shown in Fig. S2A and Fig. S2B that the examples of distributions of avalanche duration T and S on models with an average in-degree of 100 and network sizes of 0.05 and 0.2 billion, giving certain configuration parameters, are not satisfy the power law with Clauset's test ρ=0.0. In Figure S2C, we have demonstrated that the distributions of avalanche durations (T) and sizes (S) in models characterized by an average in-degree of 100 and network sizes of 5 billion, under specific configuration parameters, can be accurately fitted using a power-law distribution. The obtained exponent values are τ=1.34, and Clauset's goodness-of-fit test reports ρ=0.294 for durations and α=1.34 with Clauset's test yielding ρ=0.159 for sizes.

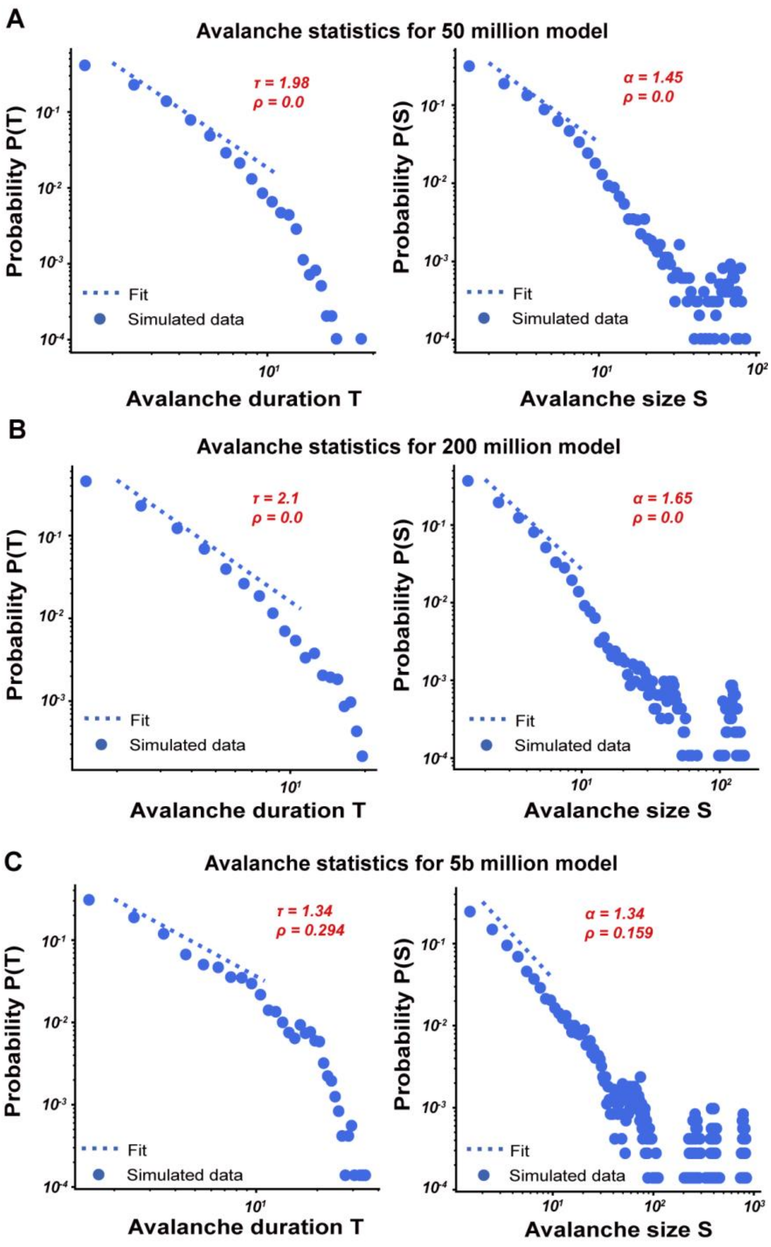

Fig. S2. Avalanche statistics in models of varying network sizes. The distribution of avalanche durations and avalanche sizes on models with an average in-degree of 100 and network sizes of 0.2, 10 and 20 billion under specific configuration parameters were illustrated in Fig. S2A, Fig. S2B and Fig. S2C, respectively.

Supplementary scaling experiments in resting states driven by interoceptive subcortices

We replicated the assimilating the resting-states under the hypothesis of being

driven by the interoceptive subcortex regions for different numbers of neurons, i.e., 0.2 billion, 10 billion and 20 billion, With the same metrics of the similarities between the assimilated model and biological counterpart in the resting-states, the mean PCCs of BOLD time course are 0.620, 0.702 and 0.709, the PCCs of the FC matrices are 0.446, 0.616 and 0.619, and the F-norm distance of the FC matrices are 0.371, 0.257 and 0.254 respectively. It is clear that the similarities increase with respect to the scale of the neurons.

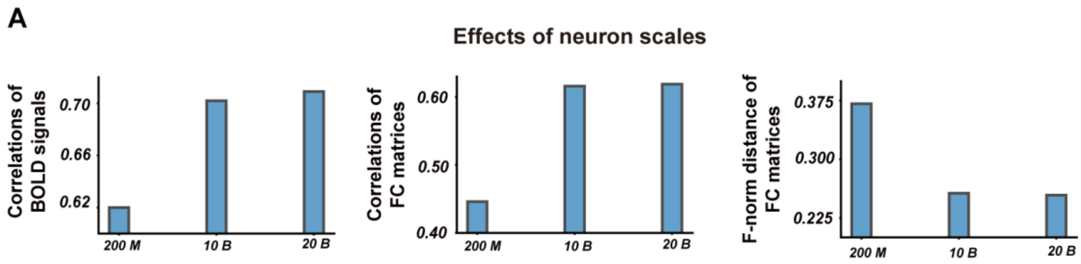

Fig. S3. Performance of the DTB in the resting state driven by interoceptive regions with respect to the number of neurons in the network. The evaluation metrics are PCC between BOLD signals, PCC between model FC and empirical FC and F-norm distance for FC matrices. One repetition of simulation experiments was conducted on models with an average in-degree of 100 and network sizes of 0.2, 10 and 20 billion.

Supplementary scaling experiments of in-degree investigation for the auditory evaluation task

We replicated the scaling experiments at the auditory evaluation experiments for the models of different numbers of neurons. For the auditory evaluation task, given the number of neurons as 0.2 billion, it is shown in Fig. S4A that the similarities between the assimilated model and biological data, measured by both the mean PCCs of the voxel-wise BOLD time courses and the PCC between the predicted scores by the model and the real scores, increases with respect to the average in-degrees.

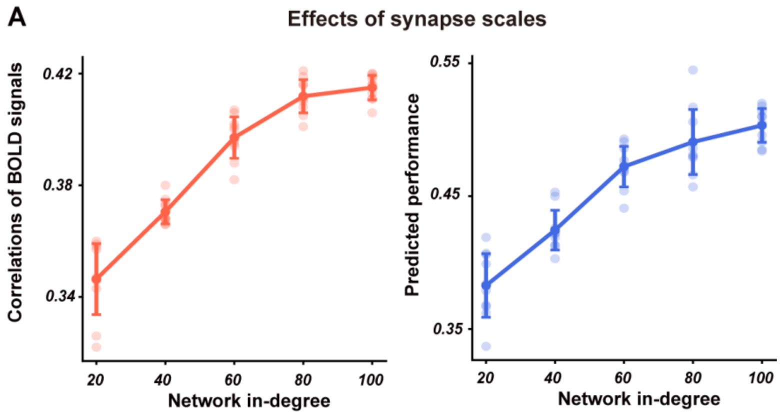

Fig. S4. Performance of the DTB for the auditory evaluation task with respect to the average in-degree. The evaluation metrics are the same as those illustrated in Figure 4B and 4C. Ten repetitions of simulation experiments were conducted on models with network sizes of 200 million and an average in-degree of 20, 40, 60, 80 and 100.

Supplementary scaling experiments for the visual evaluation task

The synaptic scaling investigations were also replicated for the visual experiments and a similar observation was held. As shown in Fig. S5A, the similarities by the same metrics used in the auditory evaluation experiments between the assimilated model and the biological subject increase with respect to the number of neurons as 0.05, 0.1, 0.2, 1, 5 and 10 billion, given the average in-degree as 100. Also, it is shown in Fig. S5B that the similarities increase with the average in-degree as 20, 40, 60, 80 and 100, given the number of neurons as 10 billion.

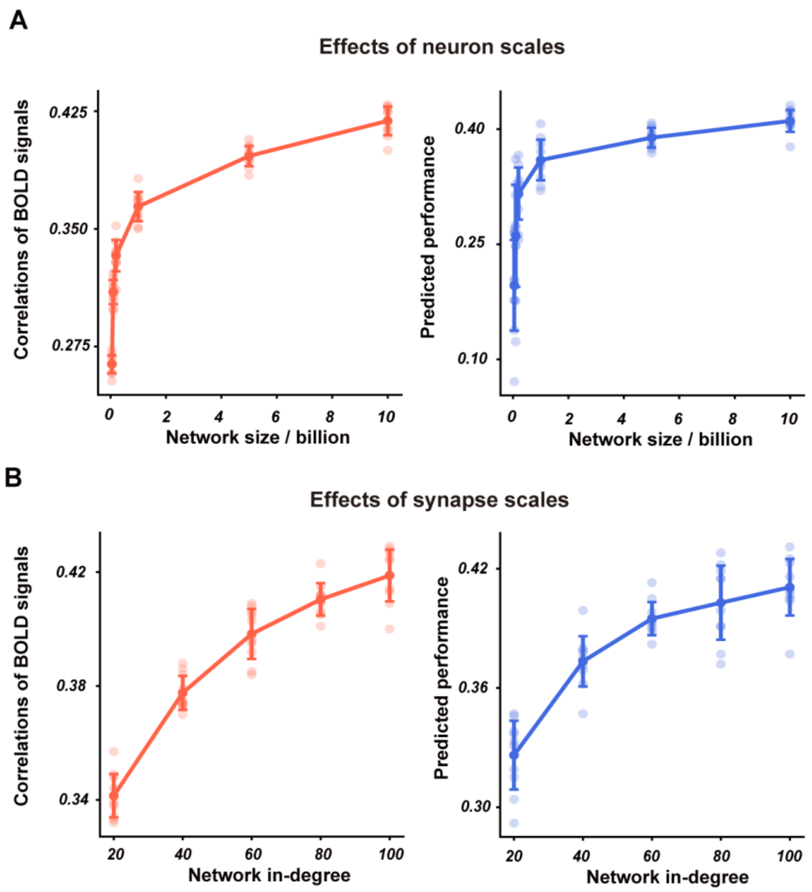

Fig. S5. Performance of the DTB for the visual evaluation task with respect to the number of neurons in the network and the average in-degree. The evaluation metrics are the same as those illustrated in Figure 4B and 4C. Upper panel: Ten repetitions of simulation experiments were conducted on models with an average in-degree of 100 and network sizes of 0.05, 0.1, 0.2, 1, 5, and 10 billion. Lower panel: Ten repetitions of simulation experiments were conducted on models with network sizes of 10 billion and an average in-degree of 20, 40, 60, 80 and 100.

Supplementary results of digital lesion operation of visual pathways

The digital lesion operation was replicated for the model of 0.2 billion neurons. As shown in Fig. S6, the mean PCC of the voxel-wise BOLD time courses at hippocampus between the assimilated model and biological data significantly decreases when removing the connections from V1 to the dorsal or/and ventral pathways (t=14.3, p-value=1e-10 for removing the dorsal; t=7.7, p-value=2e-06 for removing the ventral; t=8.9, p-value=2e-07 for removing both dorsal and ventral, where P-values were all adjusted using Bonferroni correction). However, the mean PCC of the voxel-wise

BOLD time course at the all cortex and subcortex between the assimilated model and the biological data does not significantly change after these lesion operations (all p-values>0.05).

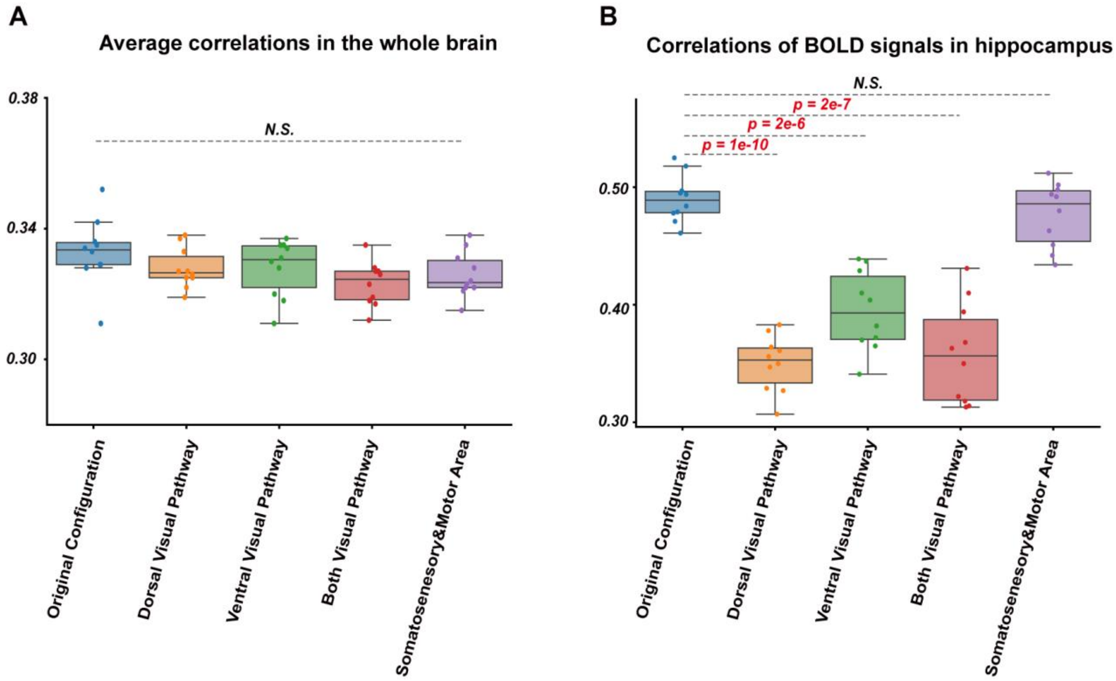

Fig. S6. Structural lesion in the visual pathways impacted the neural dynamics of the hippocampus of DTB in visual evaluation task. Ten repetitions of simulation experiments were conducted on models with network sizes of 200 million and an average in-degree of 100. **(A)** The comparisons of the average correlations of BOLD signals in the whole brain between the DTB and its biological counterpart with different lesion operations. **(B)** The comparisons of correlations of BOLD signals in the hippocampus between the DTB and its biological counterpart with different lesion operations.

Supplementary results of phase analyses

We have shown in Fig. S7A that the mean coefficient of variation (CV) of region-wise BOLD signals in the resting state across various network sizes. Our findings indicate that as the number of neurons increases from 0.05, 0.2, 1 to 5 billion, the mean CV of region-wise BOLD signals becomes more closely aligned with those of counter-part (Jianfeng Feng's) resting-state BOLD signals.

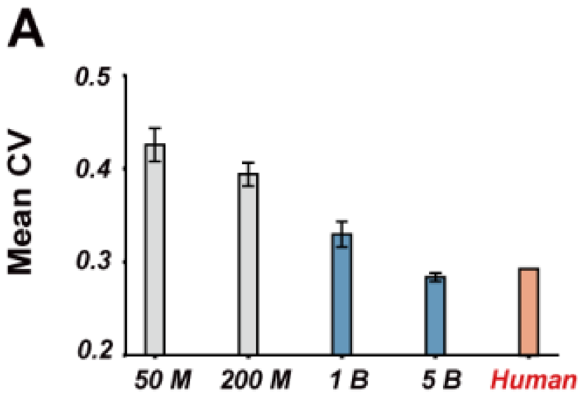

Fig. S7. Phase synchrony analysis of DTB in the resting state at various network sizes. The mean coefficient of variation of region-wise BOLD signals are calculated to quantify the variability on models with an average in-degree of 100 and network sizes of 0.05, 0.2, 1 and 5 billion, compared to the biological brain. The light gray bars correspond to the network size in the middle panel where parameters did not pass Clauset's Goodness-of-Fit test as illustrated in Fig. 2E.

Supplementary results of frequency-domain conditional Granger causality analysis of assimilated hyperparameters in interoceptive regions

To investigate the driving effects of the thalamus across different frequency bands, we also conduct frequency-domain conditional Granger causality. Our findings indicate that the thalamus predominantly exerts its influence within the respiratory and cardiac frequency bands (Fig. S8).

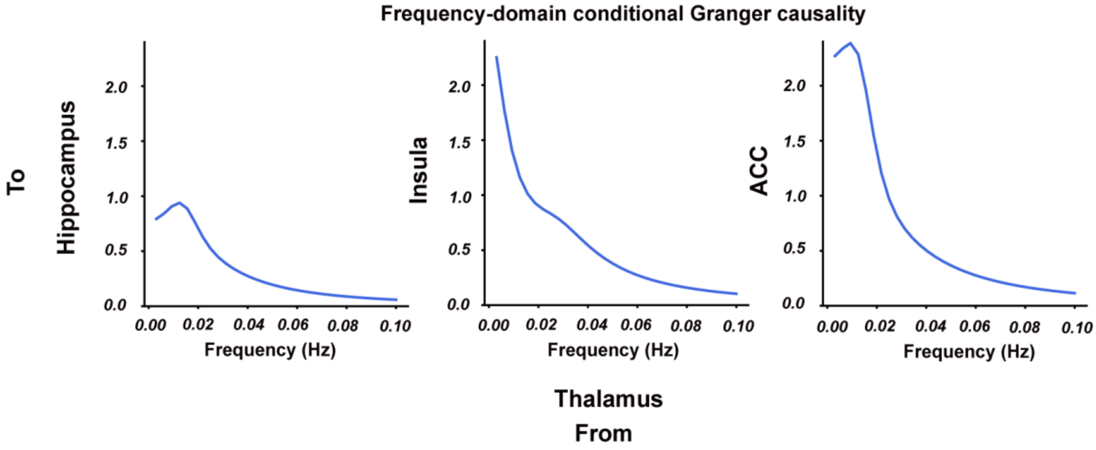

Fig. S8. Frequency-domain conditional Granger causality analysis of assimilated hyperparameters in interoceptive regions. The causal relationships from the thalamus to the hippocampus, insula and ACC are illustrated.

Supplementary results of details regarding the computational time and resources

As shown in Table 1, we present the computation resources for the models of diverse

scales and simulation performance, time-to-solution, as weak scaling experiments. The time-to-solution is measured by the average time (in msec) cost in the computation system vs. the actual biological time (in msec), where each mean firing rate of the whole network in resting states is around 2.5-3HZ. It can be seen that the time-to-solution increases with number of neurons and average in-degree. It can be also found that the time-to-solution increases with the firing rate. Data are not shown here but more details can be referred to our following preprint[21].

Table S4: Resources of the high-performance computing system and reports of the simulation performance via the time-to-solution for different scales.

Number of Neurons	Averaged in-degree	Number of GPUs for Simulation	Time-to-solution
50 million	100	5	41.5
100 million	100	10	42.0
200 million	100	20	43.2
1 billion	100	100	46.5
5 billion	100	500	52.5
10 billion	100	1000	60
20 billion	100	2000	75
1 billion	20	20	211.3
1 billion	40	40	106.3
1 billion	60	60	72.5
1 billion	80	80	56.3

Supplementary scaling experiments of in-degree investigation for the auditory evaluation task

To investigate the performance of DTB in action for the auditory evaluation task as the mean synaptic in-degree increases further, we conducted a series of simulation experiments using network sizes of 1 billion. The average in-degrees were varied to be 500, 1000, 1500, and 2000. As depicted in the figures, the similarity indices do not exhibit a consistent increase with the rise in in-degree; rather, they stabilize and tend towards saturation.

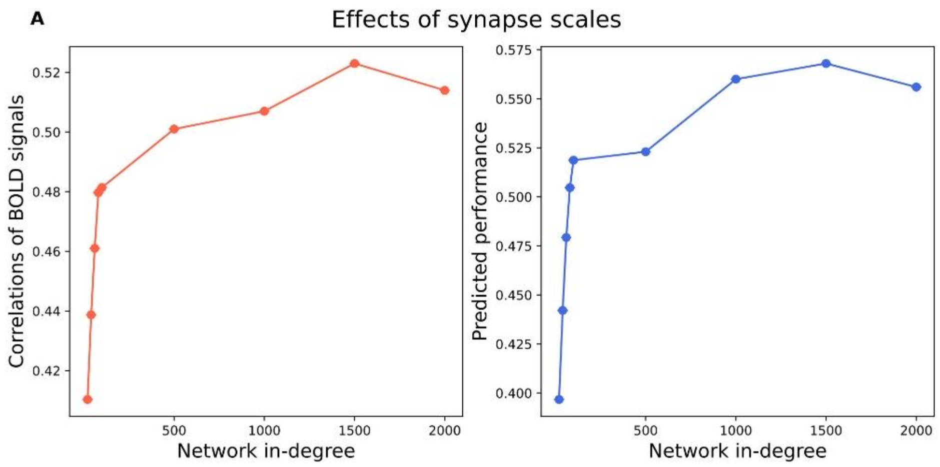

Fig. S9. Performance of the DTB for the auditory evaluation task with respect to the average in-degree. The evaluation metrics are the same as those illustrated in Figures 4B and 4C. One repetition of simulation experiments were conducted on models with network sizes of 1 billion and an average in-degree of 500, 1000, 1500 and 2000.

Supplementary experiments of the distribution of in-degree on the performance of the cortico-subcortical model

To illustrate the effect of the distribution of in-degree on the performance of our model, additional resting-state assimilation experiments on the thalamus were conducted, where the in-degree within the same neuron population followed a gamma distribution. The assimilated hyperparameter were kept consistent with the scenario of equal in-degree. Ten repetitions of each experiment were performed on the models with 1 billion neurons and an average in-degree of 100 with variances of 100, 50 and 12.5, respectively. There were no statistical differences between all conditions with a variant in-degree and the scenario of equal in-degree (two sample t-test, $p > 0.05$).

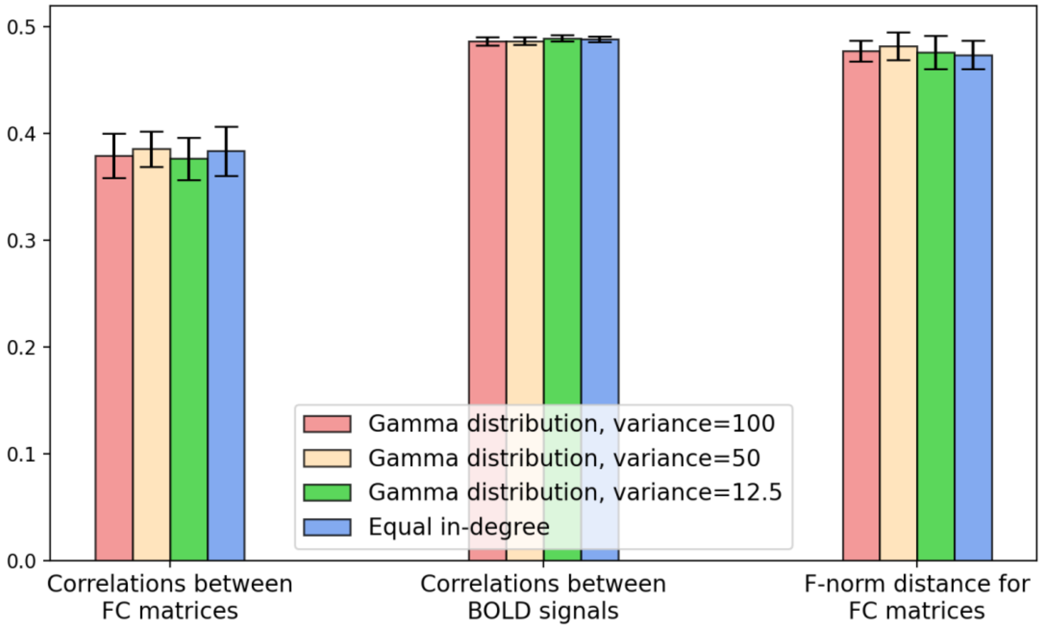

Fig. S10. Performance of the DTB in the resting state with respect to the variance of the distribution of in-degree. Ten sets of simulation experiments were conducted to evaluate the performance of DTB in the resting state. The experiments used models with a network size of 1 billion and an average in-degree of 100, with variances of 100, 50, and 12.5 in the in-degree distribution, respectively. The same three evaluation metrics as presented in Figure 2D were employed. Statistical analysis using a two-sample t-test revealed no significant differences between all conditions with varying in-degree variance and the scenario of equal in-degree ($p > 0.05$).

Supplementary experiments of the biological plausibility of the model

To demonstrate the biological rationality of our DTB model, we substituted the resting-state driving brain region with the Dorsolateral Prefrontal cortex in assimilation simulation experiments. These results were then compared with assimilation simulation results from the thalamus. Ten repetitions of simulation experiments were conducted on models with an average in-degree of 100 and network sizes of 1 billion. As illustrated in the figure, across all three metrics, the driving effect of the thalamus is significantly superior to that of the Dorsolateral Prefrontal cortex (two sample t-test, $p < 1e\text{-}16$ in all cases).

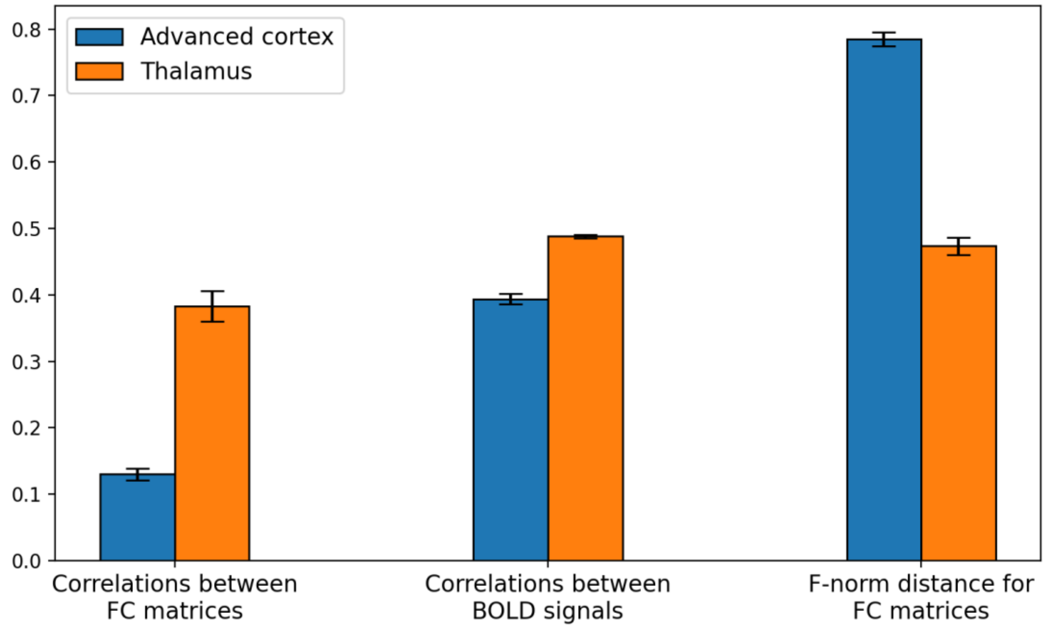

Fig. S11. Comparison of the resting states driven by the Dorsolateral Prefrontal cortex and those driven by thalamus. Ten sets of simulation experiments were conducted to evaluate the performance of DTB in the resting state. The experiments used models with a network size of 1 billion and an average in-degree of 100. The same three evaluation metrics as presented in Figure 2D were employed. The driving effect of the thalamus is significantly superior to that of the Dorsolateral Prefrontal cortex across all three metrics (two-sample t-test, $p < 1e-16$ in all cases).

Supplementary results of causal relationships of the five components in interoceptive circuits

Here we also employ Partial Cross Mapping (PCM)[22] to assess causal relationships within interoception regions. The parameters for time lags in phase-space reconstruction were set to 1, and the embedding dimensions were determined using the False Nearest Neighbors (FNN) algorithm. As depicted in the following figure, brain regions exhibiting high significance in causal relationships, such as from the thalamus to the ACC, from the thalamus to the insula, and from the thalamus to the hippocampus, as determined by conditional Granger causality, also reveal robust causal relationships using the Partial Cross Mapping method. However, it is noteworthy that the causality derived from PCM exhibits a notable symmetry, possibly influenced by the effect of "Strong Forcing," leading to the misconception of bidirectional causality in the absence thereof.

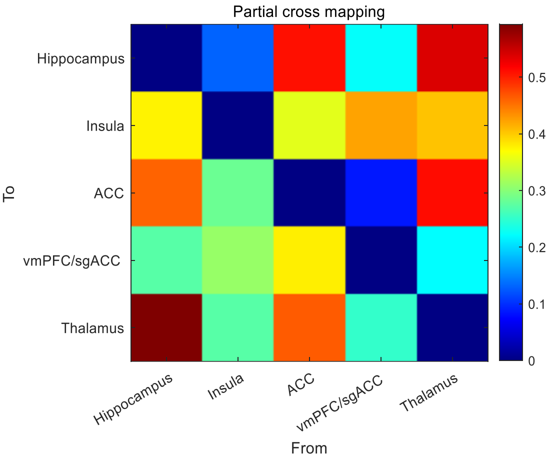

Fig. S12. C*ausal relationships of the five components in interoceptive circuits using* PCM. Brain regions demonstrating strong significance in causal relationships, such as from the thalamus to the ACC, from the thalamus to the insula, and from the thalamus to the hippocampus, identified through conditional Granger causality, also exhibit robust causal relationships using the Partial Cross Mapping method.

REFERENCES

1. Huang CC, Rolls ET, Feng JF, et al. An extended Human Connectome Project multimodal parcellation atlas of the human cortex and subcortical areas. *Brain Struct Funct* 2022; **227**: 763-78.
2. Lu W, Zheng Q, Xu N et al. The human digital twin brain in the resting state and in action. arXiv:2211.15963.
3. Binzegger T, Douglas RJ, Martin KA. A quantitative map of the circuit of cat primary visual cortex. *J Neurosci* 2004; **24:** 8441-53.
4. Du J, Vegh V, Reutens DC. The laminar cortex model: a new continuum cortex model incorporating laminar architecture. *PLoS Comput Biol* 2012; **8**: e1002733.
5. Hansen JY, Shafiei G, Vogel JW et al. Local molecular and global connectomic contributions to cross-disorder cortical abnormalities. *Nat Commun* 2022; **13**: 4682. Zeng LB, Feng JF, Lu WL. A General Description of Criticality in Neural Network Models." arXiv preprint arXiv:2309.03348 (2023).
6. Zhang W and Lu WL. Deep Diffusion Kalman Filter Combining Large-Scale Neuronal Networks Simulation with Multimodal Neuroimaging Data. *Mathematics* 2023; **11**: 2716.
7. Zhang W, Chen B, Feng J, et al. On a framework of data assimilation for hyperparameter estimation of spiking neuronal networks. *Neural Netw* 2024; **171**: 293-307.
8. Friston KJ, Mechelli A, Turner R, Price CJ. Nonlinear responses in fMRI: the Balloon model, Volterra kernels, and other hemodynamics. *NeuroImage* 2000; **12**: 466-77.
9. Phillips JW, Schulmann A, Hara E et al. A repeated molecular architecture across thalamic pathways. *Nat Neurosci* 2019; **22**: 1925-35.

10. Berntson GG and Sahib SK. Neural circuits of interoception. *Trends Neurosci* 2021; **44**: 17-28.
11. Guo S, Seth AK, Kendrick KM et al. Partial Granger causality—Eliminating exogenous inputs and latent variables. *J Neurosci Methods* 2008; **172**: 79-93.
12. Guo S, Wu J, Ding M et al. Uncovering interactions in the frequency domain. *PLoS Comput Biol* 2008; **4**: e1000087.
13. Xu L, Feng J, Yu L. Avalanche criticality in individuals, fluid intelligence, and working memory. *Hum Brain Mapp* 2022; **43**: 2534-53.
14. Tagliazucchi E, Balenzuela P, Fraiman D et al. Criticality in large-scale brain fMRI dynamics unveiled by a novel point process analysis. *Front Physiol* 2012; **3**: 15.
15. Beggs JM and Plenz D. Neuronal avalanches in neocortical circuits. *J Neurosci* 2003; **23**: 11167-77.
16. Friedman N, Ito S, Brinkman BAW et al. Universal critical dynamics in high resolution neuronal avalanche data. *Phys Rev Lett* 2012; **108**: 208102.
17. Anna D, Corral A. Fitting and goodness-of-fit test of non-truncated and truncated power-law distributions. *Acta Geophys* 2013; **61**: 1351-94.
18. Aaron C, Shalizi CR, Newman MEJ. Power-law distributions in empirical data. *SIAM Rev* 2009; **51**: 661-703.
19. Marshall N, Timme NM, Bennett N et al. Analysis of power laws, shape collapses, and neural complexity: new techniques and MATLAB support via the NCC toolbox. *Front Physiol* 2016; **7**: 250.
20. Lu WL, Zeng L, Du X et al. Digital Twin Brain: a simulation and assimilation platform for whole human brain. arXiv:2308.01241.
21. Leng S, Ma H, Kurths J et al. Partial cross mapping eliminates indirect causal influences. *Nat Commun* 2020; **11**: 2632.
